# Structure–Biofunction Relationships in Nanoparticle-Reinforced PVA/Alginate Hydrogels for Infection-Controlled Tissue-Contact Applications

**DOI:** 10.1021/acsomega.6c02968

**Published:** 2026-08-20

**Authors:** Nader Abuhamed, Emine Dinçer, Kerim Emre Öksüz

**Affiliations:** † Department of Metallurgical & Materials Engineering, 52954 Sivas Cumhuriyet University, 58140 Sivas, Türkiye; ‡ Department of Nutrition & Dietetics, Faculty of Health Science, Sivas Cumhuriyet University, 58140 Sivas, Türkiye; § Institute of Science and Technology, Department of Bioengineering, Sivas Cumhuriyet University, 58140 Sivas, Türkiye

## Abstract

Poly­(vinyl alcohol)/sodium alginate (PVA/SA) hydrogels are promising tissue-contact biomaterials; however, the comparative effects of different reinforcing nanoparticles within the same polymer matrix remain insufficiently clarified. This study aimed to compare the structural and biological performance of PVA/SA hydrogel scaffolds reinforced with zinc oxide nanoparticles (ZnONPs), hydroxyapatite (HAp), and graphene oxide nanosheets (GONs) under identical preparation conditions. PVA/SA-based hydrogels were prepared using freeze–thaw processing and evaluated by electron microscopy, EDXS, FT-IR, and Raman spectroscopy. Their swelling behavior, in vitro mass loss in PBS, antibacterial activity, L929 fibroblast cytocompatibility, hemolytic activity, and blood clotting index (BCI) were also examined. The maximum swelling ratios were 3.70 for PVA/SA, 2.46 for PVA/SA/ZnONPs, 3.39 for PVA/SA/HAp, and 3.20 for PVA/SA/GONs. After 28 days, mass loss reached 13.1% for PVA/SA, 15.9% for PVA/SA/ZnONPs, 10.5% for PVA/SA/HAp, and 10.1% for PVA/SA/GONs. The MTT assay showed acceptable cytocompatibility in all groups, with L929 cell viability of 91.74 ± 3.49% for PVA/SA, 84.35 ± 2.68% for PVA/SA/ZnONPs, 95.28 ± 2.28% for PVA/SA/HAp, and 78.55 ± 6.15% for PVA/SA/GONs after 48 h. ZnO produced the most pronounced antibacterial effect, although the activity was moderate in absolute terms, reducing *S. aureus* by 34.6% and *K. pneumoniae* by 18.5%, while other formulations showed only marginal reductions (≤7%). Hemolysis values were 3.4% for PVA/SA, 1.8% for PVA/SA/ZnONPs, 1.9% for PVA/SA/HAp, and 6.3% for PVA/SA/GONs. At 5 min, BCI values were 34.22%, 54.89%, 52.74%, and 8.7%, respectively. Overall, ZnONPs improved antibacterial activity, HAp provided the most favorable cytocompatibility and stability profile, while GONs showed a comparatively higher hemolytic response. These findings highlight the importance of nanoparticle selection in designing PVA/SA hydrogel scaffolds for tissue-contact applications.

## Graphical abstract

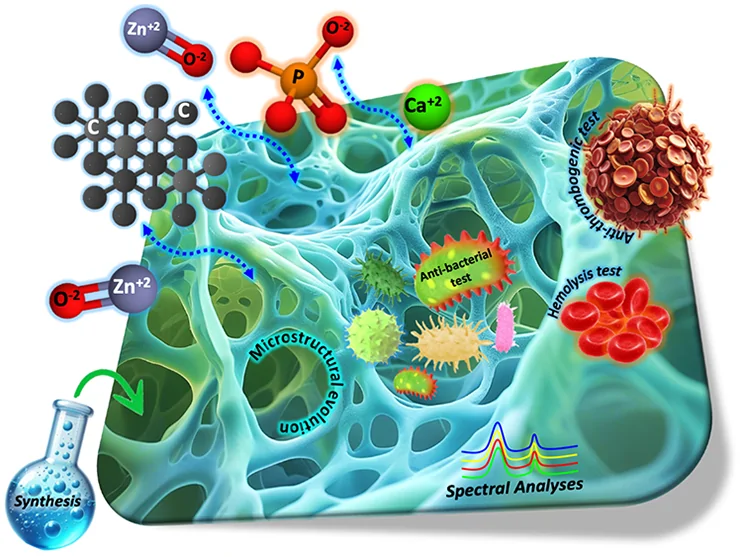

## Introduction

The process of tissue healing is multifaceted and necessitates an appropriate environment to facilitate cellular growth and regeneration. In recent years, hydrogel scaffolds have gained traction as promising materials for tissue engineering applications due to their biocompatibility, adaptable mechanical properties, and capacity to emulate the extracellular matrix (ECM). However, conventional hydrogel scaffolds often encounter limited structural stability and lower biocharacteristic, which can impede their effectiveness in fostering tissue healing.^1^

For practical translation, candidate hydrogels must satisfy multiple requirements simultaneously, including controlled fluid interaction and swelling, structural stability, degradation behavior under physiological conditions, antibacterial performance to reduce bacterial colonization, and compatibility with cells and blood.^2–4^ Balancing these properties within a simple, reproducible, and scalable fabrication route remains a major design challenge for hydrogel-based biomaterial scaffolds.^2^ Beyond these fundamental attributes, scaffold architecture, particularly porosity, pore size, and pore interconnectivity, must be carefully controlled because these features regulate cellular infiltration, migration, nutrient diffusion, oxygen transport, waste removal, and tissue formation.^5,6^ The swelling behavior of hydrogels is also important because pore structure and water uptake can influence swelling ratio, degradation rate, mechanical behavior, and molecular diffusion within the scaffold.^7^ In wound-contact systems, maintaining an appropriate moisture balance is essential, since insufficient hydration may impair the healing environment, whereas excessive moisture or fluid accumulation may promote maceration, bacterial growth, and infection risk.^8^ Furthermore, scaffold degradation should be controlled and matched with tissue remodeling, as degradation rate can influence cell proliferation, matrix remodeling, construct contraction, and neo-tissue formation.^9^ Consequently, current biomaterials research increasingly focuses on designing hydrogel synthesis and fabrication strategies that provide optimized structural, physicochemical, biological, and translational performance.^2,7^

Poly­(vinyl alcohol) (PVA) and sodium alginate (SA) hydrogel scaffolds were selected as the foundational materials because of their biocompatibility and favorable properties for tissue engineering applications.^10,11^ In particular, PVA/SA hydrogels can be prepared with tunable compositions that enable adjustment of the microstructure and transport behavior, and they have been investigated for diverse biomedical uses, including cartilage- and wound-related scaffold formats.^12–14^ Freeze–thaw processing further provides a straightforward physical cross-linking strategy to generate robust, reproducible hydrogel networks without the need for harsh chemical conditions.^15^

Nanoparticle incorporation is widely used to tune hydrogel function; however, different reinforcing agents can influence the physicochemical, mechanical, and biological properties of hydrogel composites in distinct and sometimes contradictory ways. Zinc oxide nanoparticles (ZnONPs) are frequently explored for antibacterial activity and are often incorporated into polymeric matrices to suppress bacterial growth.^16,17^ Hydroxyapatite (HAp) is a bioactive inorganic phase that can modify hydrogel microstructure and degradation behavior and is commonly associated with favorable cell–material interactions.^14^ Graphene oxide (GO) provides a high-surface-area nanosheet structure with oxygen-containing functional groups that can alter scaffold architecture and interfacial biological responses.^18^ Despite broad interest in each filler, systematic head-to-head comparisons of ZnONPs, HAp, and GO within the same PVA/SA scaffold system and under identical processing conditions remain limited, making it difficult to extract clear material-selection rules.

Recent related studies support the biomedical relevance of these material combinations. Öksüz reported that bioactive coatings on biopolymer materials can improve physical, mechanical, and in vitro properties, emphasizing the importance of surface and material modification in tissue-contact biomaterials.^19^ Abuhamed et al. investigated amoxicillin-loaded PVA/SA/ZnONPs hydrogels and reported their potential for wound healing and drug delivery applications, particularly due to their swelling behavior, biodegradation profile, antibacterial activity, and controlled drug release.^10^ Similarly, You et al. developed sodium alginate/poly­(vinyl alcohol) hydrogels containing green-synthesized ZnO nanoparticles and reported that these composite dressings showed porous structure, favorable swelling/moisturizing behavior, and antibacterial potential for wound healing applications.^20^

Although previous studies have examined PVA/SA hydrogels and individual nanoparticle-reinforced systems, direct comparative evaluation of ZnONPs, HAp, and GONs within the same PVA/SA matrix under identical preparation conditions remains limited. This gap makes it difficult to determine whether the observed changes in scaffold behavior are mainly related to nanoparticle identity or to differences in formulation and processing conditions. Therefore, a controlled comparison using the same polymer matrix, same processing route, and same nanoparticle loading is needed to better understand the structure–property–biofunction relationships of PVA/SA-based hydrogel scaffolds.

The main aim of the present study was to compare the effects of ZnONPs, HAp, and GONs on the structural, physicochemical, antibacterial, cytocompatibility, and hemocompatibility properties of PVA/SA hydrogel scaffolds prepared under identical conditions. The novelty of this work lies in the direct comparison of three different reinforcing nanoparticles within the same PVA/SA scaffold system, allowing the influence of nanoparticle type to be evaluated more clearly. This comparative approach provides useful guidance for selecting suitable nanoparticle-modified PVA/SA hydrogel scaffolds for tissue-contact biomedical applications.

## Materials and Methods

### Chemicals

Nanopowders, GO, and hydrogel synthesis were performed with analytical grade, potassium permanganate (KMnO_4_), graphite powder (C), sodium hypochlorite (NaOCl), sodium chloride (NaCl), sodium nitrate (NaNO_3_), sodium hydroxide (NaOH), glutaraldehyde, (OCH­(CH_2_)_3_CHO, 25% aqueous solution,), EtOH, (CH_3_CH_2_OH, nondenatured ethanol), zinc acetate dihydrate [Zn­(CH_3_COO)_2_·2H_2_O, ≥99%], ((CH_3_)_2_CHOH), ACS reagent, ≥99.5%), hydrochloric acid (HCl, 30% (*w*/*v*), sulfuric acid (H_2_SO_4_, 37% (v/v)), hydrogen peroxide (H_2_O_2_, 30% (w/w) in H_2_O) solution, poly­(vinyl alcohol) polymer (PVA-(C_2_H_4_O)­x; Mw = 60.000 g/mol), sodium alginate ((SA, (C_6_H_7_O_6_Na); viscosity = ≥2,000 cP, 2% (25 °C, lit.) were purchased from Sigma-Aldrich (St. Louis, USA). Additional analytical-grade in vitro reagents and materials were sourced from Merck KGaA (Darmstadt, Germany), Thermo Fisher Scientific (Massachusetts, USA), and Bayer AG (Leverkusen, Germany)

### Production of HAp

In accordance with our previous research^21,22^ HAp were obtained with some modifications from natural bovine bones using the following method. Briefly, freshly collected bone parts were thoroughly cleaned, boiled six times in dH_2_O, and degreased with 70% EtOH. These bone pieces were then placed in NaClO (30% v/v) and left to dry for further processing, taking care that they were allowed to dry for at least 48 h. Next, the dried bone pieces were calcined at a temperature of 850 °C with a heating rate of 5 °C/min and kept at that temperature for 2 h in the air to eliminate any prions, such as Creutzfeldt–Jakob disease (CJD) or Bovine Spongiform Encephalopathy (BSE).^23^ Following that, the calcined bovine femoral bone parts were crushed into small pieces and subjected to planetary ball milling (The Fritsch Planetary Micro Mill Pulverisette 7 premium line, Germany) in isopropyl alcohol using ZrO_2_ balls at 600 cycles/min for 48 h (ball to powder ratio of 10:1) and then dried in a Pyrex pan. Finally, the natural HAp were stored at room temperature (RT) in a desiccator (Figure 1a).

**Figure 1:**
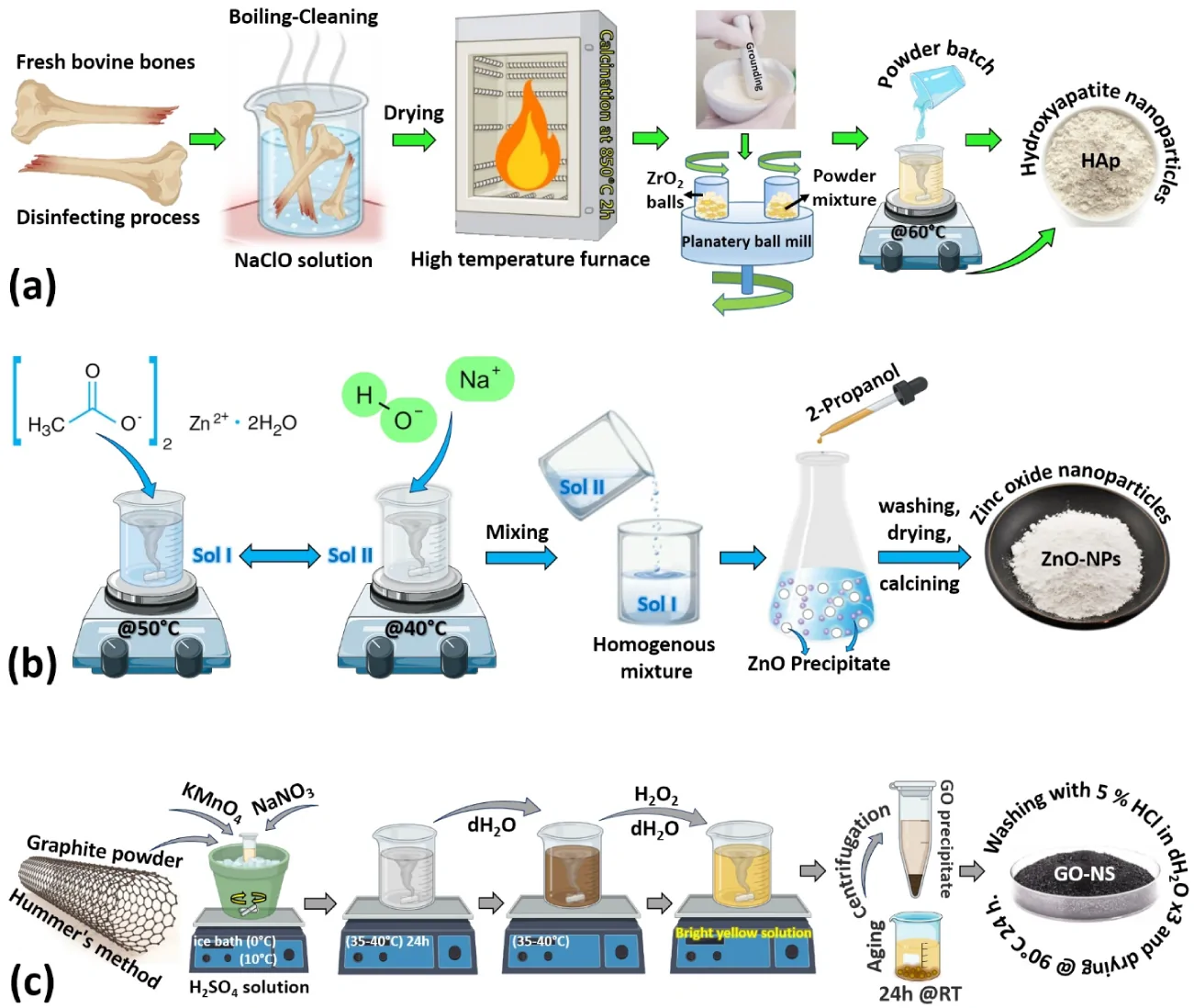
The layout of the HAp, ZnONPs and GONs synthesis steps and methodologies. (**a**) Flowchart of natural HAp derived from fresh bovine bones. (**b**) Chemical steps of ZnONPs synthesis. (**c**) Controlled chemical synthesis of large GONs via Hummers’ method. Certain schematic elements in this figure were provided by Servier Medical Art (https://smart.servier.com), licensed under CC BY 4.0 (https://creativecommons.org/licenses/by/4.0/).

### Synthesis of ZnONPs

ZnONPs were synthesized by a sol–gel method per the literature^24^ with minor modifications. In this procedure, 2 g of Zn­(CH_3_COO)_2_·2H_2_O was dissolved in 15 mL of dH_2_O, while 8 g of NaOH was dissolved in 10 mL of dH_2_O. The solutions were stirred for 30 min each before the NaOH solution was added to the Zn­(CH_3_COO)_2_ solution with constant magnetic stirring for 15 min. 2-Propyl alcohol was then gradually added drop by drop (1 mL/300 s.) to the mixture, causing a white precipitate to form. The resulting nano ZnO precipitate was washed several times with dH_2_O to eliminate any residues. Following washing, the ZnO nanoparticles were dried at 80 °C in a hot air oven and then heated in an oven at 700 °C for 4 h,^25^ resulting in the production of pure white ZnO nanopowder (Figure 1b).

### Synthesis of GONs

A modified Hummer’s method was utilized to synthesize graphene oxide nanosheets (GONs) from pure graphite powder.^26^ Around 2 g of graphite powder was stirred in 40 mL of H_2_SO_4_ in an ice bath for 15 min. Subsequently, KMnO_4_ and NaNO_3_ were slowly added to the solution while keeping the temperature below 10 °C. The mixture was stirred for 6 h and allowed to react in a water bath at 35–40 °C for 24 h. The solution was diluted with 70 mL of dH_2_O and stirred until it turned brown. Further dilution was done by adding another 110 mL of dH_2_O. The mixture was then treated with a mixture of H_2_O_2_ and dH_2_O to convert the residual permanganate and MnO_2_ into soluble MnSO_4_. This step resulted in a bright yellow solution. The remaining mixture was centrifuged and washed thrice with 5% HCl in dH_2_O to remove any residue. GO was obtained by drying the solid sample in an oven at 90 °C for 24 h (Figure 1c). A detailed schematic of the synthesis steps and procedures used to obtain both nanoparticles and GONs were provided in Figure 1.

### Preparation of HAp-ZnONPs-GONs Reinforced Hydrogel Scaffolds

A 9% (w/v) PVA solution was prepared by dissolving PVA in ddH_2_O at 80 °C with constant stirring for 4 h. Separately, SA was dissolved in ddH_2_O at 60 °C to obtain a 1% (w/v) SA solution. The two were then combined at 60 °C in a 9:1 volume ratio to form the PVA/SA solution. For polymer/nanoparticle compositions to produce different hydrogels, HAp, ZnONPs, and GONs powders were each dispersed in ddH_2_O by sonication using an ultrasonic homogenizer (SONICS, VCX/750, Ultrasonic processors, Newtown, U.S.A.) for 15 min at 0.1% (w/v). The boundary amount of 0.1 wt % was chosen mainly because of the clustering or agglomeration of nanoparticles at higher concentrations.^27^ A fixed nanoparticle loading of 0.1 wt % was selected to enable direct comparison among ZnONPs, HAp, and GONs while minimizing concentration-dependent effects such as aggregation, reduced dispersion uniformity, and possible interference with hydrogel network formation. This low loading was also selected from a safety-oriented perspective, since excessive filler incorporation may increase cytotoxic or hemolytic risks, particularly for nanoscale inorganic and carbon-based fillers. In the present study, even at 0.1 wt %, the biological responses differed among fillers: PVA/SA/GONs showed lower L929 viability than the other groups and higher hemolysis, whereas ZnONPs and HAp remained nonhemolytic. Therefore, the same low loading was used for all fillers to isolate the effect of filler identity rather than concentration while maintaining acceptable short-term biological compatibility. After dispersion, HAp, ZnONPs, and GONs suspensions were separately added to the prepared PVA/SA solution. Then the oxide solutions were dispersed in the resulting dispersion by sonication at 40 ± 2 °C for 15 min, followed by mechanical mixing for 1 h to prepare PVA/SA/HAp, PVA/SA/ZnONPs, PVA/SA/GONs polymer solutions. To prepare the various oxide reinforced hydrogel scaffolds, the mixture was first allowed to stand for 12 h to remove air bubbles. The freeze–thaw cycle (F-T) method was then used to prepare the scaffolds. Polymer matrix solutions in different compositions, which were prepared by mixing separately, were poured into Petri dishes. The mixtures were frozen at a temperature of −20 °C and then subsequently lyophilized at a temperature of −55 °C (Labconco Freezone Lyophilizer, U.S.A.) for 24 h. This process was repeated for two consecutive cycles. Two freeze–thaw cycles were selected to obtain physically cross-linked PVA/SA scaffolds while maintaining a porous morphology suitable for swelling and tissue-contact evaluation. Increasing the number of freeze–thaw cycles may increase crystallite formation and physical cross-link density, but it can also reduce water uptake and alter pore structure. Therefore, two cycles were used as a balanced processing condition for comparing the three types of reinforcing agents under identical scaffold preparation parameters. After the second cycle, the hydrogel scaffolds were thawed at room temperature for 6 h, consistent with previously reported freeze–thaw processing approaches for PVA-based scaffold systems.^15^ The resulting hydrogel scaffolds were sterilized using gamma irradiation,^28^ to ensure that they were both sterile and cross-linked. Control hydrogel scaffolds were also prepared using the same procedure without the addition of oxide reinforcement. All the hydrogel scaffolds were then stored in plastic bags at a temperature of 4 °C for later use.

### Morphological and Elemental Composition Evaluation

Nanoparticle and nanosheet morphologies were examined by FE-SEM (Tescan Mira3 XMU) operated at 15–25 kV. Samples were mounted on carbon tape conductive film and coated with gold using an ion sputter coater (SCM-200, Polaris, Republic of Korea). Surface composition was analyzed by EDXS (INCA IE 350, U.K.) attached to the FE-SEM. For S-TEM imaging, powder dispersed in solution was loaded onto a carbon coated copper grid (carbon type-B, 300 mesh copper grids, Ted Pella, Inc., Redding, CA, USA) and dried at 40 °C.^29^

### Fourier-Transform Infrared Spectroscopy (FT-IR)

FT-IR was used to assess the chemical and physical properties of the synthesized hydrogels. Spectra for the materials were collected on a Spectrum One from Perking Elmer (Bruker Alpha II FT-IR, USA) operating in attenuated total internal reflection mode. For each spectrum, four scans were acquired over 4000 cm^–1^ to 400 cm^–1^ with a resolution of 4 cm^–1^. Measurements were taken at RT and analyzed in OriginPro version 6.0 (OriginLab).

### Raman Spectroscopy

The Raman spectra of the synthesized hydrogels were recorded using a Renishaw in Via Raman Microscope. Measurements were carried out using excitation helium–neon lasers with wavelengths of 532 and 785 nm. The recorded spectra covered a Raman shift range from 400 cm^–1^ to 4000 cm^–1^, as defined by the experimental setup. Data acquisition was performed using an advanced CCD array comprising 1024 × 256 pixels. To ensure optimal performance, the CCD array was cooled by a Peltier device and kept at −70 °C. To eliminate elastically scattered laser light, a notch filter was added. The laser power was carefully controlled at 0.085 mW/μm^2^ over a 20 μm^2^ area. The spectra were obtained at RT and further analyzed using Wire 5.0 software program to identify characteristic peaks and shifts of the hydrogels. In all spectral analyses, prepared lyophilized hydrogels were randomly selected in two pairs.

### In Vitro Biodegradation

In vitro biodegradation is often used to assess the biocompatibility or biodegradability of materials, such as hydrogels used in medical or other applications. The hydrogels were weighed after being cut into equally sized pieces (1 × 1 × 1 cmcube shape). For 1, 7, 14, 21, and 28 days, they were in phosphate-buffered saline solution (PBS) at 37 °C. The in vitro experiments were carried out in triplicate (*n = 3*) for 1, 7, 14, 21, and 28 days. The hydrogels were taken out, washed with dH_2_O, and dried after each time. Using a digital scale, the samples’ weight was determined before and after incubation. The difference in weight before and after the incubation time was used to compute the weight loss ratio. The weight loss ratio was calculated according to^30^ as indicated below (eq 1):

1 $$\mathrm{Weight}\;\mathrm{loss}\;\mathrm{ratio}=\frac{W_{1}-W_{2}}{W_{1}}\times 100\%$$

where *W*_1_ and *W*_2_ represent the weights of the hydrogels before and after in vitro biodegradation, respectively.

### Swelling Ratio of Hydrogel Scaffolds

The prepared hydrogels were weighed and placed in a PBS solution with a pH of 7.4 at RT for various periods. At regular intervals, the weight of the patches was determined. The filter paper was used to absorb any extra surface water, and this procedure was continued until the sample achieved saturation. The following formula (eq 2) was then used to determine the swelling ratio (SR) of hydrogel samples:^31^

2 $$\mathrm{SR}=\frac{W_{t}-W_{d}}{W_{d}}$$

where *W*_*t*_ represents the weight of the swelling hydrogel sample and *W*_d_ stands for the weight of the dry hydrogel sample. These variables are used in a formula to calculate the swelling ratio of the hydrogel samples as a percentage.

### Cytotoxicity Test

Cell viability was assessed using an MTT reduction assay with L929 fibroblast cells, following previously reported cell-culture procedures with minor modifications. Hydrogel samples were cut into 0.5 × 0.5 cm specimens and sterilized under UV light at 260 nm for 30 min. L929 cells were cultured in DMEM supplemented with 10% fetal bovine serum at 37 °C in a humidified 5% CO_2_ atmosphere.^19^ Sterile hydrogel specimens were placed in 48-well plates, and 1 mL of L929 cell suspension was added to each well at a cell density of 1 × 10^4^ cells/mL to allow direct exposure of the cells to the hydrogel samples. After 48 h of incubation, 0.5 mL of MTT solution was added to each well, and incubation was continued for 4 h. Subsequently, 1 mL of DMSO was added to dissolve the formed formazan crystals, and absorbance was measured at 490 nm using a microplate photometer reader (Thermo Scientific Multiskan FC, U.S.A.).^32^ Cell viability was calculated relative to the untreated control group. Each composition was tested in triplicate, and the results were reported as mean ± standard deviation. L929 cell morphology after 48 h of exposure to the hydrogels was examined using a 100× inverted microscope.

### Antithrombogenic Properties

Antithrombogenicity was assessed by measuring blood clotting index (BCI). The procedure followed Patil et al.^33^ and Bohorquez-Moreno et al.^34^ with minor modifications. Goat blood was collected at a slaughterhouse into a tube containing sodium citrate (C_6_H_5_Na_3_O_7_) and used immediately. For testing, 0.25 g of each sample (∼0.5 × 0.5 cm) was placed at the center of Petri dishes, 100 μL fresh blood was applied to the surface, and 10 μL of 0.2 M CaCl_2_ was added immediately. Samples were incubated at 37 °C for 5, 10, 15, and 20 min. After incubation, 5 mL sterile dH_2_O was gently added without disturbing the clot. The blood–dH_2_O mixture was collected, transferred to falcon test tubes, centrifuged at 10.000 rpm for 1 min, and the supernatants were moved to clean tubes and incubated at 37 °C for 1 h. Absorbance was measured at 540 nm against a sterile dH_2_O blank using BMG LABTECH’s ultrafast UV/vis spectrometer (SPECTROstar Nano, Germany). For the positive control (+), 5 mL sterile dH_2_O and 100 μL blood were used. Clotting capacity was confirmed twice per sample, and the mean of three independent experiments (n = 3) was used to compute BCI. BCI was calculated using (eq 3) given below:

3 $$\mathrm{BCI}=\left(\frac{{\mathrm{OD}}_{s}}{{\mathrm{OD}}_{\mathrm{PC}}}\right)\times 100$$

where OD_s_ denotes the tested sample absorbance and OD_PC_ denotes the positive control (+) absorbance.

### Hemocompatibility Test

Hemolytic activity was assessed using a modified method described by Wang et al. Fresh blood was collected, RBCs were separated by centrifugation, washed 3× with PBS, and diluted 10-fold with PBS. Then 1 mL of the diluted RBCs and 25 mg of test sample were mixed in a sterile polypropylene tube and incubated at 37 °C for 1 h. After incubation, the mixture was centrifuged for 1 min at 10.000 rpm, and supernatants were collected for spectroscopic analysis. Optical density (OD) of supernatants was measured at 541 nm using BMG LABTECH’s ultrafast UV/vis spectrometer (SPECTROstar Nano, Germany). A positive control (+) consisting of a mixture of 0.8 mL sterile dH_2_O and 0.2 mL diluted RBCs and a negative control (−) consisting of a mixture of 0.8 mL PBS and 0.2 mL diluted RBCs were also used.^35^ The hemolytic activity percentage (%) was determined (eq 4) by performing the experiment in triplicate and averaging the results.

4 $$\mathrm{Hemolytic}\;\mathrm{activity}=\left(\frac{{\mathrm{OD}}_{S}-{\mathrm{OD}}_{\mathrm{NC}}}{{\mathrm{OD}}_{\mathrm{PC}}-{\mathrm{OD}}_{\mathrm{NC}}}\right)\times 100\%$$

where OD_S_ is the absorbance of the tested sample, OD_NC_ is the absorbance of the (−) control and OD_PC_ is the absorbance of the (+) control. Hemolysis is classified by absorbance as >5% (hemolytic), 2–5% (slightly hemolytic), or 0–2% (nonhemolytic).

### Antimicrobial Evaluation

This study aimed to evaluate the effectiveness of test samples in preventing the growth of bacteria using a modified version of a method introduced by Gao et al.^36^ In the study, Mueller Hinton Broth (MHB) medium was created, and test samples were added and sterilized. Indicator microorganisms including one Gram-positive *Staphylococcus aureus*, (ATCC 29213) and four Gram-negative *Escherichia coli*, (ATCC 25922), *Klebsiella pneumonia*, (ATCC 700603), *Pseudomonas aeruginosa*, (ATCC 27853) and *Proteus vulgaris*, (ATCC 7829) bacteria were used as indicators of the test samples’ effectiveness. The indicator microorganisms were grown in 10 mL MHB for 16–24 h, and their densities were adjusted according to McFarland No 0.5. They were then diluted by 1/100 and 200 μL of inoculum were added to 1.8 mL of modified MHB, containing the test samples. Then the mixture was incubated for 24 h at 37 °C. Viable colony counts were performed on MHB using the plate counting method. Samples were serially diluted 10-fold in buffered peptone water, and 100 μL of each dilution was spread onto MHA plates. Plates were incubated at 37 °C for 24 h, then viable cells were enumerated. Each assay was run twice, and the mean of the two repetitions was used for calculations. Results were evaluated using (eq 5):

5 $$\mathrm{Bacteria}\;\mathrm{reduction}=\frac{\log{\mathrm{CFU}}_{\mathrm{Control}}-\log{\mathrm{CFU}}_{\mathrm{Treatment}}}{\log{\mathrm{CFU}}_{\mathrm{Control}}}\times 100\%$$

where log CFU_control_ and log CFU_Treatment_ are the numbers of colony-forming units of the control sample and the treated sample, respectively.

### Statistical Analyses

Data are reported as mean ± SD from at least three independent measurements (*n = 3*). Statistical analysis used one-way ANOVA followed by the Bonferroni *t* test. Significance was set at *p ≤ 0.05. All analyses were performed in OriginPro 9.0 software program.

## Results and Discussion

### Nanoparticles Morphology

The S-TEM micrographs of the HAp, ZnONPs and GONs particles are shown in Figure 2. The particle size and morphology of ZnONPs, HAp and GONs analyzed by S-TEM and represented in Figure 2, reveal that most of the ZnONPs nanoparticles are quasi-spherical with a diameter of approximately 30–40 nm and the average particle size of HA nanocrystals is approximately 90–100 nm. The resultant nanoparticles are porous in nature, as seen in the S-TEM analysis. The S-TEM images of HAp show particles with an irregular morphology, transitioning between spherical and needlelike shapes. S-TEM images of HAp and ZnONPs reveal partial agglomeration, likely due to their high specific surface area and surface energy. The S-TEM image of GONs illustrates their flake-like shapes, and the texture of the nano sheets can be observed in the images. GONs shows a distinctive, transparent, and distorted laminar structure, with evident surface undulations and rough edges. The undulations and rough edges are the result of reaction kinetic phenomenon resulting from reduction and oxidation processes during the production of the GO powders. The microstructural observations indicate that the internal structure of the prepared GONs possesses a folded morphology. The presence of undulating folds and rough edges in the carbon sheets is evidence of the successful synthesis of GO sheets, as previously noted by several authors.^37,38^

**Figure 2:**
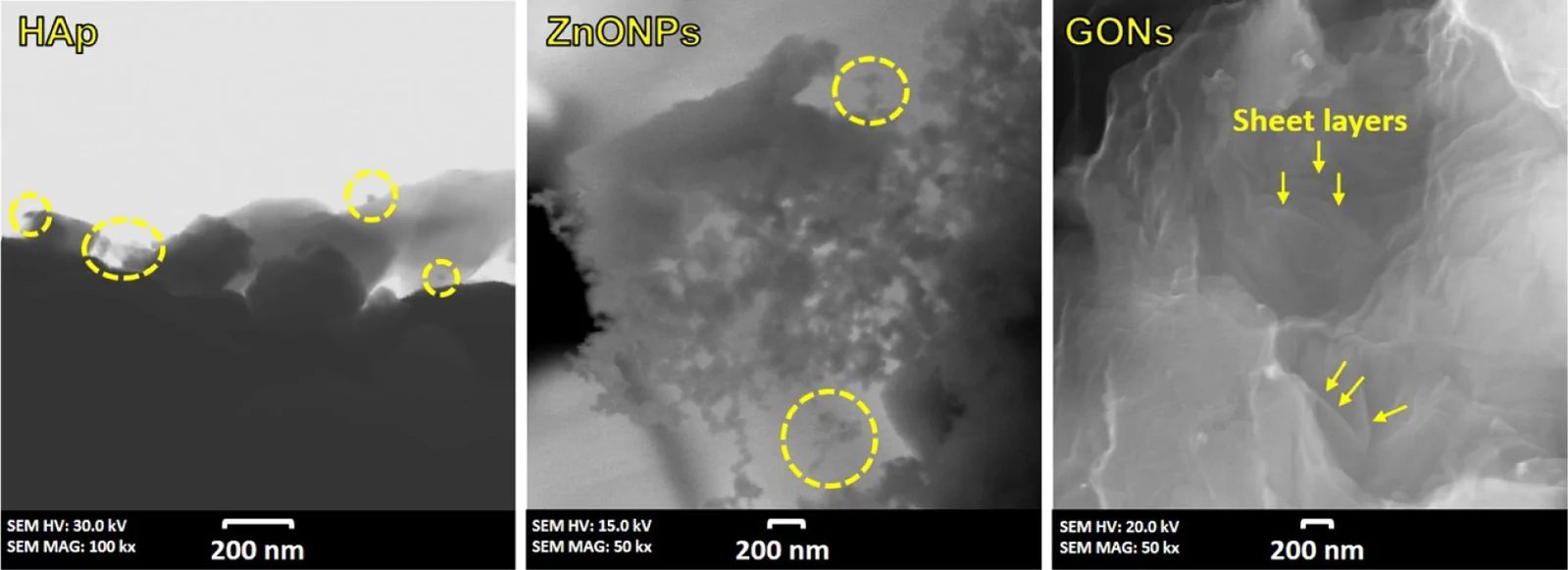
S-TEM micrographs of the synthesized HAp, ZnONPs and GONs at different magnifications.

### Hydrogel Scaffolds Morphology

Figure 3 presents the FE-SEM photos of the PVA/SA hydrogel scaffolds synthesized in this study, incorporating various fillers such as ZnONPs, HAp, and GONs. The observed porous structure across all hydrogels suggests successful gelation during the freeze–thawing cycles. Interestingly, the PVA/SA hydrogel exhibited larger surface voids, porosity and surface roughness compared to the other hydrogels containing fillers. Upon closer examination, the hydrogel scaffolds containing GONs and ZnONPs presented smoother surfaces, with the GO-infused hydrogel exhibiting few pores. In contrast, the hydrogel incorporating HAp displayed a higher degree of porosity on its surface. The porosity observed in the FE-SEM micrographs supports the swelling behavior results shown in Figure 7a. The PVA/SA and PVA/SA/HAp hydrogel scaffolds demonstrated the highest swelling rates among all tested samples. The presence of microporosity in these biomaterials facilitates hydrogel swelling by enabling more accessible H_2_O molecule transport and reducing diffusion resistance. These findings suggest that the distinct surface morphology and porosity of PVA/SA-based hydrogel scaffolds, as revealed by FE-SEM analysis, play a crucial role in their swelling behaviors. Understanding these properties is essential for optimizing hydrogel performance in various applications, such as drug delivery systems and tissue engineering biomaterial scaffolds. Although the SEM observations are qualitative, the filler-dependent differences in surface morphology and apparent porosity support the idea that HAp and GONs altered the network architecture, which may have limited water penetration and delayed mass loss during PBS incubation.

**Figure 3:**
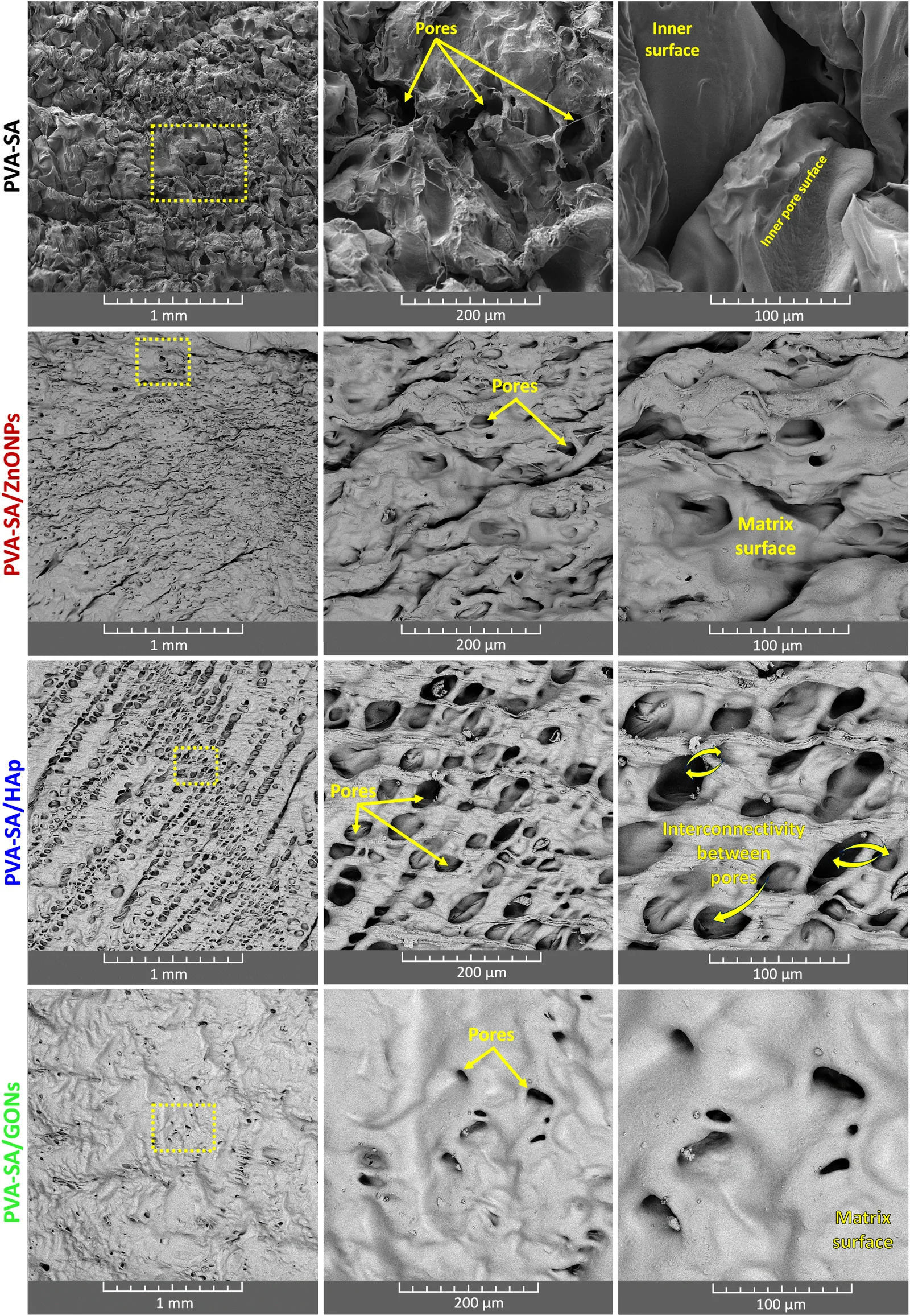
FE-SEM micrographs of surface morphology of PVA/SA incorporated with ZnONPs, HAp, and GONs synthesized hydrogel scaffolds. For each hydrogel scaffold, photos at progressively higher magnifications are presented from left to right. Pore morphologies, pore interconnections, and matrix surfaces observed in the surface images of the synthesized hydrogel scaffolds are indicated with yellow marks.

### EDXS Analyses

The EDXS analysis of the PVA/SA-based hydrogels in Figure 4 provides detailed information on the elemental composition and structural modifications introduced by the incorporation of ZnONPs, HAp, and GO. The EDXS spectrum of the pure PVA/SA hydrogel reveals peaks corresponding to carbon (C), oxygen (O), and sodium (Na). The C and O peaks are attributed to the organic nature of PVA and SA, while the Na signal originates from the SA component. The PVA/SA/ZnONPs hydrogel spectrum displays additional peaks corresponding to Zn, confirming the successful incorporation of ZnONPs into the hydrogel matrix. In the case of the PVA/SA/HAp hydrogel, the EDXS spectrum reveals prominent peaks for phosphorus (P) and calcium (Ca) alongside the C, and O peaks. These elements are characteristic of HAp and confirm its incorporation into the hydrogel structure. The PVA/SA/GONs hydrogel spectrum demonstrates a significant increase in the C signal, consistent with the high C content of GO. This confirms the successful integration of GONs into the hydrogel matrix. Overall, the incorporation of ZnONPs, HAp, and GONs into the PVA/SA hydrogel matrix results in distinct changes in both elemental composition and surface morphology.

**Figure 4:**
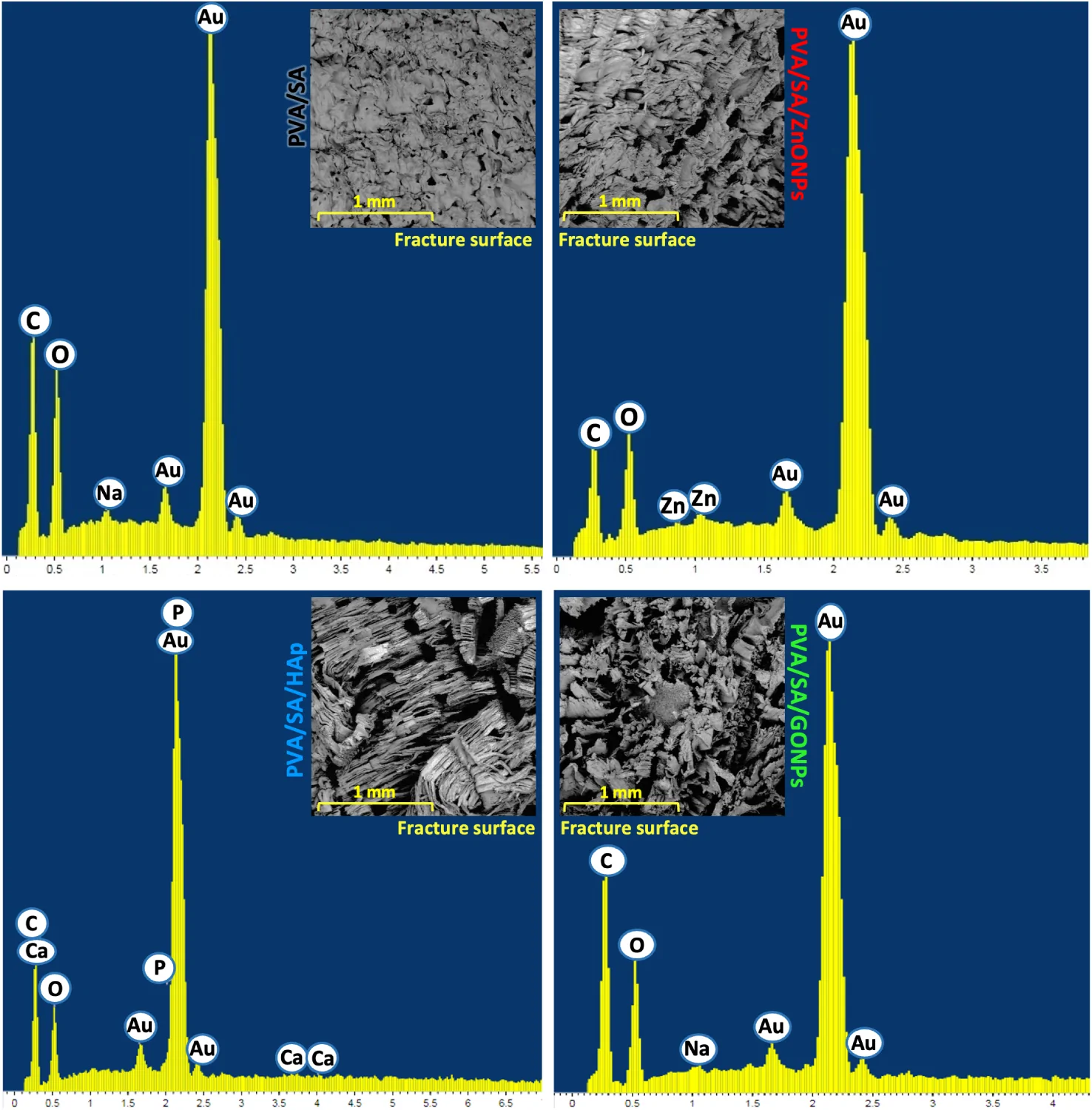
EDXS analyses of hydrogel scaffolds on fracture surfaces.

### FT-IR Analyses

FT-IR spectroscopy was used to identify characteristic functional groups and possible interactions among PVA, SA, and the incorporated nanoparticles/nanosheets. FT-IR of the PVA/SA combined with ZnONPs, HAp, and GONs were measured in the region of 500–4000 cm^–1^. In order to understand the existence of the integrated components, FT-IR analysis is essential. Figure 5 displays the spectra of PVA/SA, PVA/SA/ZnONPs, PVA/SA/HAp, and PVA/SA/GONs hydrogel scaffolds. The PVA/SA spectrum displays a broad O–H stretching band at 3280 cm^–1^, reflecting H-bonding between PVA and SA hydroxyls.^39^ −H stretching bands at 2916 and 2852 cm^–1^ arise from alkyl groups. Due to residual acetate groups in PVA, a carbonyl stretching peak appears at 1731 cm^–1^.^40^ A CH_2_ deformation is observed at 1410 cm^–1^. Peaks at 1610 and 1419 cm^–1^ are assigned to the asymmetric and symmetric stretches of alginate carboxylate (COO^–^) groups.^41^ SA cyclic ether linkage gives a strong band at 1373 cm^–1^,^39^ while the C–O stretching accounts for the 1089 cm^–1^ absorption;^42^ a slight shoulder at 1026 cm^–1^ indicates R–O stretching of the terminal SA bond.^43^

**Figure 5:**
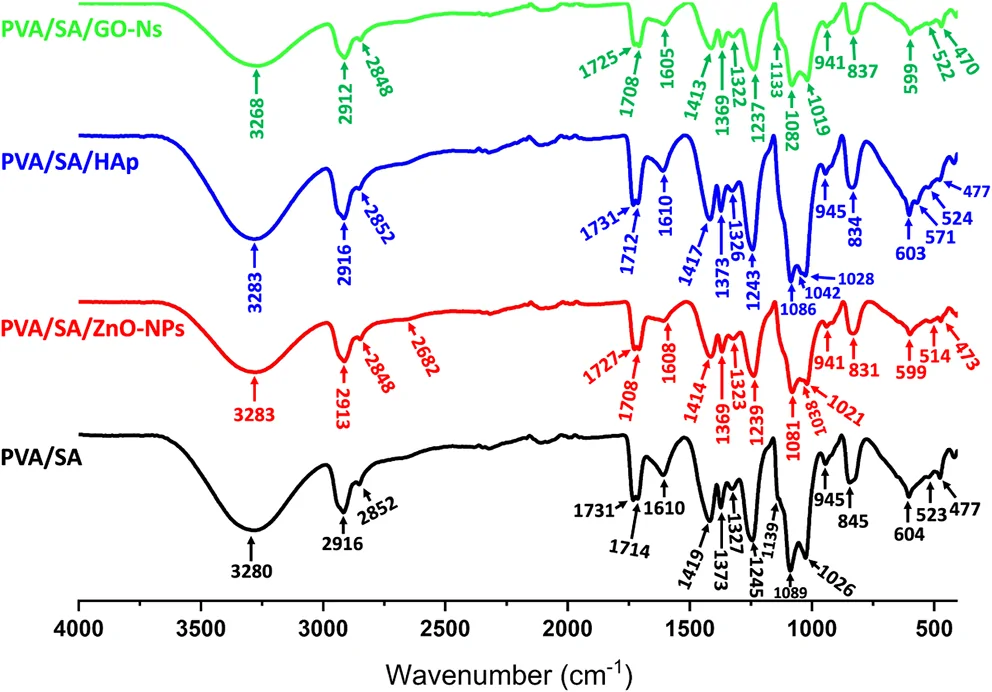
FT-IR spectra of all hydrogel scaffolds. (Color arrows highlight regions containing specific peaks).

In PVA/SA/ZnONPs hydrogels, modest shifts in the O–H and C=O bands suggest interactions between ZnO and the PVA/SA matrix. A peak at 514 cm^–1^ (Zn–O stretching) confirms ZnO within the PVA complex, and symmetric wavenumber shifts with decreased band intensities relative to PVA/SA further indicate specific network interactions.^44^ For PVA/SA/GONs, the characteristic C–O–C stretching bands at 1237 and 1082 cm^–1^ are attributable to GO.^45^ GO reinforcement led to a shift toward lower wavenumbers, particularly in the O–H/–C–OH stretching region (3100–3500 cm^–1^). This shift is consistent with disruption of the original PVA O–H hydrogen bonds and the formation of new hydrogen-bonding interactions among PVA, SA, and GONs.^46–48^

A new band at 571 cm^–1^ corresponds to the symmetric stretch of PO_4_^3–^ indicating HAp in the PVA/SA hydrogel; its presence also denotes HAp free of organic residues. The absence of organics in HAp is attributed to high-temperature calcination (>700 °C). Additionally, the increased intensity at 603 cm^–1^ in PVA/SA/HAp versus PVA/SA is consistent with HAp–hydrogel interactions.^49^ The observed peak shifts and intensity changes suggest possible hydrogen bonding, filler–matrix interactions, and changes in the local polymer chain environment after nanoparticle incorporation.

### Raman Spectroscopy

Raman spectroscopy analysis in Figure 6 reveals several characteristic bands for PVA/SA hydrogels. The band observed at 919 cm^–1^ is attributed to the deformation vibrations of the O–H bond in the composite material.^50^ The peak at 1444 cm^–1^ is linked to the stretching vibrations of the −CH groups in PVA molecules,^51^ while the prominent band centered around 2918 cm^–1^ corresponds to the stretching vibrations of −CH_2_ groups.^52^ Additionally, the peak at 1353 cm^–1^ represents C–O stretching vibrations, and the band at 1092 cm^–1^ corresponds to the glycosidic ring breathing mode in SA.^53^ Furthermore, the Raman spectra of PVA/SA hydrogels display a band at 836 cm^–1^, which corresponds to C–O–C stretching vibrations.^54^ These observations provide a clear baseline for the structural characteristics of the PVA/SA hydrogel matrix. The Raman spectra of the hydrogels show significant differences based on the incorporated nanoparticles or nanosheets, highlighting the impact of each additive on the vibrational and structural properties of the PVA/SA matrix. The addition of ZnONPs, HAp, or GONs not only introduces new spectral features but also causes noticeable shifts in the main matrix spectra, reflecting the chemical interactions and structural modifications achieved through these additives. When ZnONPs are incorporated into the PVA/SA hydrogel, notable changes in the Raman spectra occur. The multiphonon scattering modes are identified at 503 cm^–1^, 648 cm^–1^, and 1075 cm^–1^, corresponding to the vibrational modes E_1_(TO) + E_2L_, 2­(E_2H_ – E_2L_), and A_1_(TO) + E_1_(TO) + E_2L_, respectively. Furthermore, the acoustic combination modes involving A_1_ and E_2_ are observed at approximately 1101 cm^–1^.^55,56^ These peaks indicate the successful incorporation of ZnONPs into the hydrogel matrix and support their contribution to the vibrational features of the composite. Similarly, the incorporation of HAp into the PVA/SA hydrogel results in distinct Raman spectral features. The symmetric stretching mode of the phosphate PO_4_^3–^ion is observed at 963 cm^–1^, while the asymmetric *v*_3_ vibrations of PO_4_^3–^ produce a band at 1066 cm^–1^.^57^ These characteristic peaks confirm the presence of HAp in the hydrogel and suggest its role in enhancing the bioactivity and structural stability of the composite. The addition of GONs to the PVA/SA hydrogel introduces two prominent peaks in the Raman spectra: The D peak at 1357 cm^–1^ and the G peak at 1598 cm^–1^. The D peak reflects the degree of disorder in the crystal structure and is associated with the sp^3^ hybrid mode of carbon atoms, while the G peak corresponds to the first-order scattering E_2_g vibration mode linked to the sp^2^ hybrid mode of carbon atoms.^47^ These peaks demonstrate the successful incorporation of GONs into the hydrogel and their influence on the structural and network stability of the composite.

**Figure 6:**
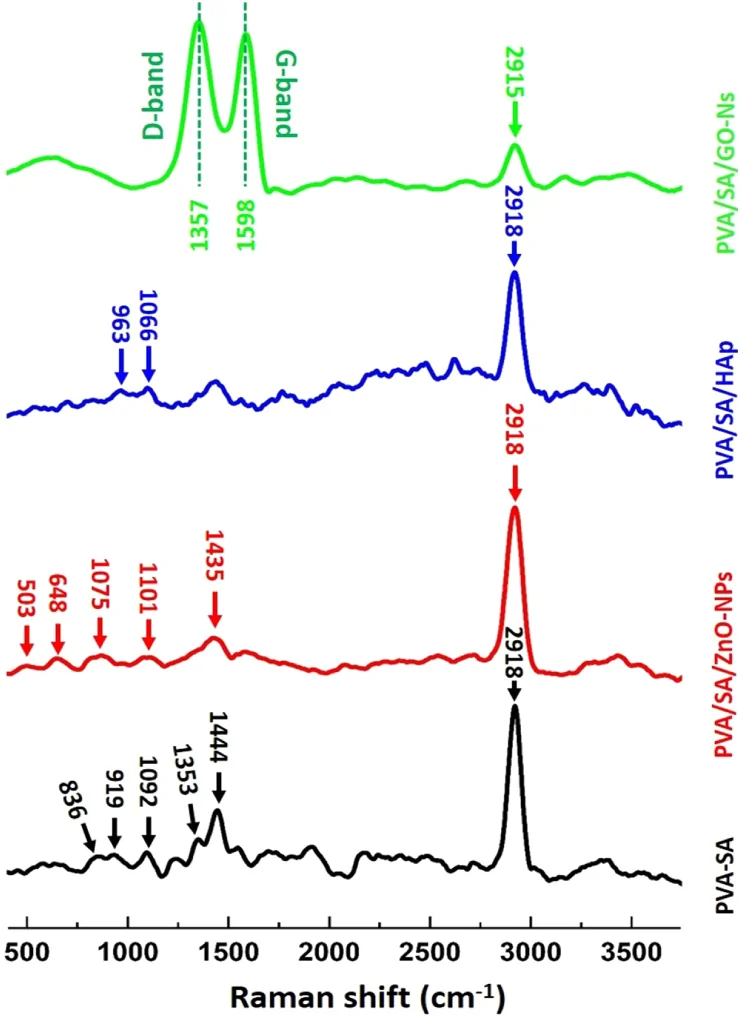
Raman spectra of all hydrogel scaffolds.

### Swelling Behavior

The high-water retention capacity of hydrogel membranes makes them essential for many biological processes, including wound healing and antibacterial characteristics. They have a large capacity for absorbing fluid from wounds, which quickens the healing process. A hydrogel’s chemical composition, including the presence of hydrophilic groups, the density of cross-links, the flexibility of the polymer network, as well as the pH and temperature of the surrounding environment, affects how much water hydrogels can store. In essence, a hydrogel’s structure and chemistry influence swelling behavior.^58^ The results of the study on the impact of filler content on the swelling behavior of PVA/SA blend hydrogels are shown in Figure 7a. It can be observed that the samples exhibit varying levels of swelling. The swelling ratio for PVA/SA increased during the first 24 h, after which it leveled off. The maximum swelling ratios for PVA/SA, PVA/SA/ZnONPs, PVA/SA/HAp, and PVA/SA/GONs were 3.70, 2.46, 3.39, and 3.20, respectively. It is well established that an increase in cross-link density produces a more compact network, leaving less free volume for water uptake in the polymer matrix; consequently, the swelling ratio is inversely related to cross-linking density. The reduced swelling observed after ZnONPs incorporation may indicate a more restricted polymer network or partial occupation of free volume within the hydrogel structure. This interpretation is consistent with the FE-SEM morphology and FT-IR spectral changes, which suggest filler–matrix interaction. Because PVA is more hydrophilic than SA, PVA/SA blend hydrogels exhibit a relatively high equilibrium swelling ratio, which is consistent with the high PVA content, in agreement with the findings of Xie et al.^59^

**Figure 7:**
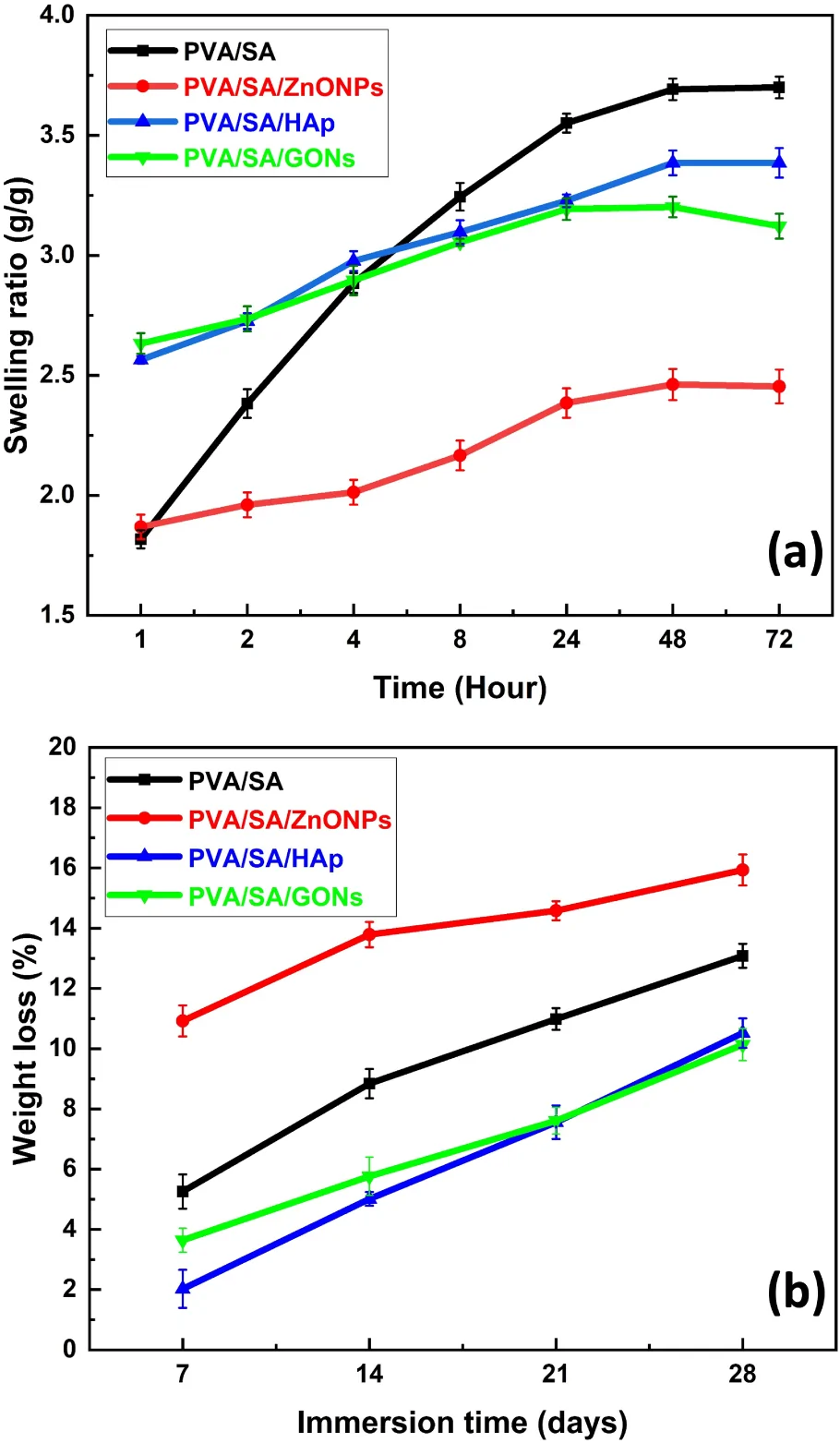
Swelling ratio (a) and in vitro biodegradation (b) behavior of hydrogel scaffolds in PBS (pH ∼ 7.4 at 37 °C). Error bars signify the standard deviation (±SD) observed for three independent experiments (*n* = 3).

Moreover, the incorporation of ZnO into PVA/SA decreased the swelling ratio of the hydrogels. This is likely due to ZnO filling some of the empty spaces within the hydrogel network and hindering the penetration of water, which reduces the expansion.^60^ Similarly, adding ZnO to starch/gelatin nanocomposite film improved its hydrophobicity and water resistance.^61^ Furthermore, the swelling ratio of PVA/SA/GONs and PVA/SA/HAp during the first 4 h showed clear differences compared with PVA/SA alone. The presence of HAp can markedly influence the swelling behavior of PVA/SA. Several studies have reported that increasing HAp content raises the crystallinity of the composite and decreases its degradation rate; at the same time, higher HAp concentrations are associated with a lower swelling ratio^62,63^ Other reports indicate that incorporation of nano-HAp into PVA/SA enhances mechanical strength and slows down swelling, owing to the reinforcing effect of HAp crystals within the polymer network.^64,65^ In addition, integration of HAp has been shown to reduce hydrophilicity, and embedding HAp into the PVA/SA matrix renders the hydrogels stiffer and less capable of absorbing liquid, as documented in.^66^ Nevertheless, the hydrophilic groups present on HAp surfaces improve cell–material adhesion and increase the biological activity of the surface.^67^

### In Vitro Biodegradation

Figure 7b represents the results of an in vitro biodegradation test for the prepared all hydrogel scaffolds. The biodegradation of each material is measured in terms of weight loss, expressed as a percentage, over a period of 4 weeks. In an in vitro biodegradation test, a sample of the material to be tested is placed in PBS solution. The sample is then monitored over time to measure the rate and extent of biodegradation. In this case, the weight loss of the samples is used as a proxy for biodegradation. The results of the test show that all four materials undergo some degree of biodegradation over the four-week period. PVA/SA had the lowest average weight loss of 5.25% ± 0.56 after 7 days, increasing to 13.08% ± 0.39 after 28 days. PVA/SA/ZnO had the highest average weight loss, with 10.92% ± 0.51 after 7 days, increasing to 15.93% ± 0.51 after 28 days. PVA/SA/HAp had an average weight loss of 2.02% ± 0.63 after 7 days, increasing to 10.51% ± 0.48 after 28 days. PVA/SA/GO had an average weight loss of 3.64% ± 0.39 after 7 days, increasing to 10.13% ± 0.53 after 28 days.

The PVA/SA hydrogel scaffold showed the second-highest degradation rate. This can be attributed to disruption of polymer chain connectivity within the hydrogel, arising from the reduced cross-linking density associated with the presence of SA, as previously reported.^68^ The high water solubility of SA further contributes to overall weight loss of the hydrogels. In addition, most of the blended components that were not effectively cross-linked but merely entrapped in the gel network degraded rapidly, which explains the progressive increase in the slope of the degradation curves for all samples over time.^69^ ZnONP incorporation was associated with the highest mass loss among the reinforced hydrogels, and it was notably higher than the other two nanoparticles used. In the FE-SEM micrographs ZnONPs had modified the porosity and surface area of the PVA-based hydrogel scaffolds. This modification leads to higher porosity and a larger surface area; this can increase the accessibility of water and other degradation agents to the hydrogel matrix, promoting hydrolytic degradation and, consequently, increasing the biodegradation rate.^3^ When it comes to the PVA/SA/GONs sample, the degradation was the lowest among the hydrogel samples after 4 weeks, as GO is known to improve the network stability of PVA.^70^ PVA/SA/HAp showed the lowest degradation rate in the first 21 days. This could be due to proper interactions between HAp and PVA and SA chains, forming strong bonds between the hydrogel matrix and the HAp. These interactions can lead to a more compact network structure, thus reducing the biodegradation rate of the hydrogel.^71^

The lower mass loss observed for PVA/SA/HAp and PVA/SA/GONs compared with PVA/SA/ZnONPs may be associated with improved physical stabilization of the polymer network via nanoparticle–matrix interactions. This interpretation is consistent with the FT-IR results, where the PVA/SA/HAp scaffold showed phosphate-related bands around 571 and 603 cm^–1^, supporting HAp incorporation and possible interaction with the polymer matrix,^49^ while the PVA/SA/GONs scaffold showed GO-related C–O–C bands and shifts in the O–H/–C–OH peak region, suggesting new hydrogen-bonding interactions between GO oxygen-containing groups and PVA/SA chains.^45–48^ Similar alginate–hydroxyapatite systems have shown phosphate–carboxylate interactions by FT-IR^49^ and Raman^57^ spectroscopy and embedded HAp within porous scaffolds by FE-SEM, supporting the role of HAp in improving scaffold properties.

The combined FE-SEM, FT-IR, Raman, swelling, and degradation results suggest that each nanoparticle modified the PVA/SA hydrogel network through a different mechanism. In the PVA/SA/ZnONPs scaffold, FE-SEM showed a more restricted morphology compared with the neat PVA/SA scaffold, while FT-IR showed shifts in the O–H and C=O regions together with the Zn–O band at 514 cm^–1^, indicating interactions between ZnONPs and hydroxyl/carboxyl-containing segments of the PVA/SA matrix.^44^ Although PVA/SA/ZnONPs exhibited the lowest swelling ratio, its higher 28-day mass loss suggests that reduced water uptake does not necessarily indicate greater long-term stability. The lower swelling may be explained by ZnONPs occupying free volume and interacting with hydroxyl- or carboxyl-containing regions of the PVA/SA network, thereby restricting initial polymer chain expansion. However, prolonged PBS exposure may also be affected by nanoparticle distribution within the matrix. In ZnO-containing polymer systems, ZnO content has been reported to promote bead-like structures and nonuniform morphology because ZnO nanoparticles agglomerate with polymer solutions.^72^ Therefore, in the present PVA/SA/ZnONPs scaffold, partial ZnONP agglomeration or locally nonuniform nanoparticle distribution may have created heterogeneous water-accessible regions, facilitating PBS-mediated matrix erosion during prolonged incubation.

In contrast, the PVA/SA/HAp scaffold showed relatively open porosity by FE-SEM and phosphate-related FT-IR bands at approximately 571 and 603 cm^–1^, together with Raman phosphate vibrations at 963 and 1066 cm^–1^, confirming HAp incorporation and suggesting mineral–polymer interactions.^49,57^ Mechanistically, HAp may act as an inorganic reinforcing phase that preserves accessible pore channels for water uptake while stabilizing the surrounding PVA/SA chains through interfacial interactions; this explains why PVA/SA/HAp maintained relatively high swelling but showed lower mass loss than PVA/SA/ZnONPs.

For PVA/SA/GONs, FT-IR showed GO-related C–O–C bands and shifts in the O–H/–C–OH region, while Raman confirmed the characteristic D and G bands of GO, supporting the incorporation of oxygen-functionalized nanosheets capable of hydrogen bonding with PVA/SA chains.^45–48^ These GO–polymer interactions, together with the two-dimensional nanosheet geometry, can reduce polymer chain relaxation, increase diffusion path tortuosity, and act as a physical barrier against PBS penetration and polymer erosion. This mechanism explains the reduced swelling and the lowest mass loss observed for PVA/SA/GONs. Therefore, the different swelling and degradation profiles in Figure 7 are not only consequences of pore morphology observed in FE-SEM, but also arise from nanoparticle spectroscopic evidence of ZnONPs–polymer, HAp–polymer, and GONs–polymer interactions that alter the internal network architecture of the PVA/SA scaffold.

### In Vitro Cytocompatibility

The cytotoxicity of biomaterials is a critical parameter in biomedical applications and is typically assessed using colorimetric assays such as MTT or XTT. In this study, the MTT assay was employed to evaluate cell viability across five experimental groups: Control, PVA/SA, PVA/SA/ZnONPs, PVA/SA/HAp, and PVA/SA/GONs, using L929 fibroblast cells cultured in 48-well plates. Figure 8 presents microscopic photos of the hydrogel samples, along with a quantitative representation of cell viability determined via the MTT assay. The results indicate that all hydrogel formulations supported cell proliferation, with cell viability exceeding 78.5%. Among the tested formulations, the highest cell viability was observed in the PVA/SA/HAp (95.28 ± 2.28%), PVA/SA (91.74 ± 3.49%), and PVA/SA/ZnONPs (84.35 ± 2.68%) groups. However, the PVA/SA/GONs group demonstrated a slightly lower cell viability (78.55 ± 6.15%), indicating a minor reduction in cell proliferation. After 48 h of exposure to the various experimental groups, L929 cells exhibited a fusiform morphology on the culture plates. This elongated spindle-shaped appearance indicated that the cells remained metabolically active and had successfully adapted to the surface.^73^ Furthermore, the majority of the tested groups demonstrated robust proliferation and growth. These findings suggest that none of the synthesized groups induced significant cytotoxic effects on L929 cells following 48 h of incubation. Moreover, the high biocompatibility of the tested groups facilitated strong cell adhesion to the hydrogel surfaces.

**Figure 8:**
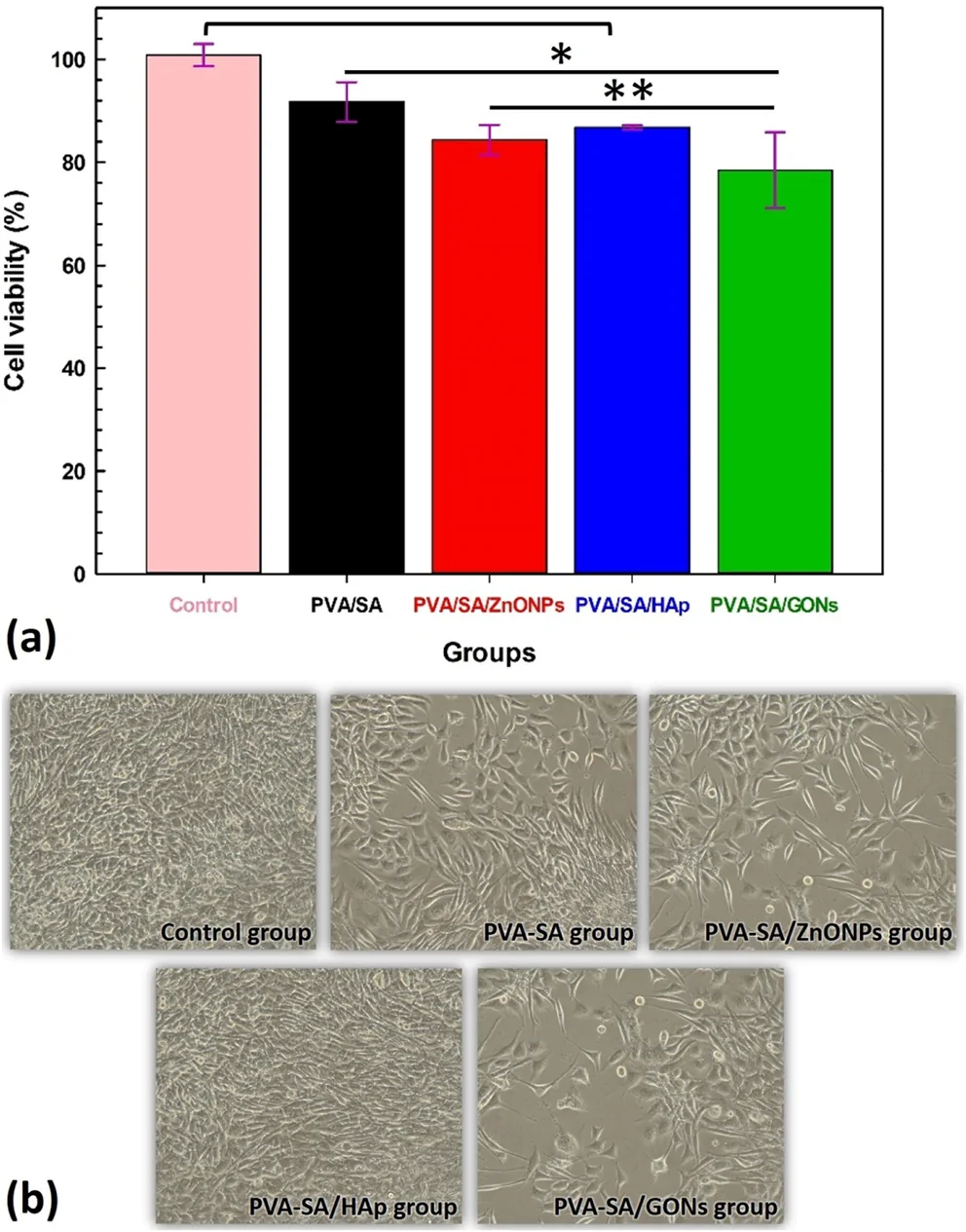
In vitro cytocompatibility evaluation of PVA/SA-based hydrogel scaffolds using L929 fibroblast cells after 48 h of exposure. (a) Quantitative cell viability (%) determined by the MTT assay for the control, PVA/SA, PVA/SA/ZnONPs, PVA/SA/HAp, and PVA/SA/GONs groups. (b) Representative optical microscopy images showing the morphology and distribution of L929 cells after exposure to the control, PVA/SA, PVA/SA/ZnONPs, PVA/SA/HAp, and PVA/SA/GONs groups. Values are presented as mean ± SD from triplicate analyses (*n* = 3). Statistical significance is indicated as (*p < 0.05 and **p < 0.001).

### Microbiological Evaluations

Hydrogels are a class of polymeric materials that are swollen with water or biological fluids, they have many potential applications in medical and biological fields due to their biocompatibility, biodegradability, and the ability to mimic the ECM. One of the most important properties of hydrogels is their antimicrobial activity, which is critical for preventing the growth and spread of microorganisms on the surface of the hydrogel. In this study, the antimicrobial properties of hydrogels were evaluated against several bacterial strains, including *P. aeruginosa*, *P. vulgaris*, *E. coli*, *K. pneumoniae*, and *S. aureus*. Compared with previously reported ZnO-containing antibacterial hydrogel systems, these reduction values indicate a moderate rather than strong.^20,74,75^ The moderate antibacterial response may also be related to the low nanoparticle loading used in the present study (0.1 wt %), which was intentionally selected to minimize aggregation and possible biological risks. The results in Table 1 show that among the four formulations tested, the PVA/SA/ZnONPs hydrogel scaffold showed the most pronounced antibacterial response among tested hydrogels, with a reduction of 34.6% against *S. aureus*, 18.5% against *K. pneumoniae* and 6.49% against *E. coli* microorganisms.

**Table 1:** Antibacterial Activity Data of the Hydrogels

Microorganism	PVA/SA/ZnONPs	PVA/SA	PVA/SA/GONs	PVA/SA/HAp
*P. aeruginosa*	5.66%	4.57%	–	–
*P. vulgaris*	3.7%	–	–	2.4%
*E. coli*	6.49%	–	–	–
*K. pneumoniae*	18.5%	–	–	3%
*S. aureus*	34.6%	7.2%	6.4%	3.6%

ZnO is a well-known antimicrobial agent that has been used for many years in various forms such as powders, nanoparticles, and films. Hydrogel reinforced ZnONPs can release Zn^2+^, which are toxic to bacteria. ZnO has been discovered to have antibacterial properties due to its semiconducting nature. The low band gap of 3.31 eV, along with its antimicrobial, UV-resistant, and photocatalytic properties, contributes to its effectiveness against bacteria. When the ZnO absorbs energy from an incident ray, the valence band electron becomes excited and jumps to the conduction band, creating an electron hole. This hole, along with the excited electron, reacts with bacteria absorbed on the surface of the ZnO and damages the cell wall, ultimately killing the bacteria. The addition of ZnO leads to an increase in antibacterial activity against Gram-positive bacteria, such as *S. aureus*. However, the antibacterial activity of ZnO is lower against Gram-negative bacteria, such as *E. coli* and *P. vulgaris*, due to their thicker cell wall which resists ZnO more effectively.^72^

PVA/SA hydrogel scaffold had the second-highest antibacterial activity with a reduction of 7.2% against *S. aureus*, 4.57% against *P. aeruginosa*. PVA is a water-soluble polymer that has been used in various applications such as wound dressings, drug delivery systems, and tissue engineering. PVA hydrogel can physically entrap bacteria, preventing them from growing. Hydrogel reinforced GONs showed a less effective antimicrobial activity with a reduction of 6.4% against *S. aureus*. GO is a material derived from graphene and composed of a single layer of carbon atoms bonded together in a hexagonal lattice. PVA/SA/GONs hydrogel scaffold has a high surface area, which can increase the adsorption of bacteria.^76^ Hydrogel reinforced HAp showed a less effective antimicrobial activity with a reduction of 3% against *K. pneumoniae*, 2.4% against *P. vulgaris*. HA is a natural mineral that is present in bones and teeth and has been used in various applications such as bone replacement, dental implants, and tissue engineering. HAp has a high surface area, which can increase bacterial adsorption. Furthermore, HAp is a (Ca_3_(PO_4_)_2_)-based bioceramic material that can release calcium Ca^2+^ and phosphate PO_4_^3–^ ions upon dissolution in an aqueous environment. The release of these ions can create an unfavorable environment for bacterial growth. High concentrations of Ca^2+^ and PO_4_^3–^ ions can interfere with bacterial cell membrane stability and enzymatic activities, leading to a bacteriostatic or bactericidal effect.^77^

In the microbiology tests, bacterial solutions were added to the PVA/SA-based hydrogel scaffolds containing ZnONPs, HAp, and GONs. After incubating these hydrogels at 37 °C for 24 h, the appearance of the plates showing bacterial viability is presented in Figure 9. Based on the obtained findings, after the PVA/SA/ZnONPs hydrogel scaffolds, which caused the highest bacterial mortality, were kept at room temperature for 24 h, the presence of bacterial colonies was observed using FE-SEM photographs of the hydrogels with and without ZnONPs. High magnification images of the hydrogels without ZnONPs showed bacterial colonies within the structure (Figure 9c). In contrast, no bacterial colonies were observed in the examined FE-SEM fields of the ZnONP-containing hydrogel (Figure 9d). These results provide evidence that the PVA/SA/ZnONPs hydrogel scaffolds are particularly effective against *S. aureus* microorganisms. Overall, the results of the study demonstrate that PVA/SA/ZnONPs hydrogel scaffold has the highest antimicrobial activity among the hydrogels tested, followed by PVA/SA hydrogel scaffold, while PVA/SA/GONs and PVA/SA/HAp hydrogel scaffolds showed less effective antimicrobial activity. These findings suggest that PVA/SA/ZnONPs and PVA/SA hydrogel scaffolds may be useful where moderate antibacterial support is required.

**Figure 9:**
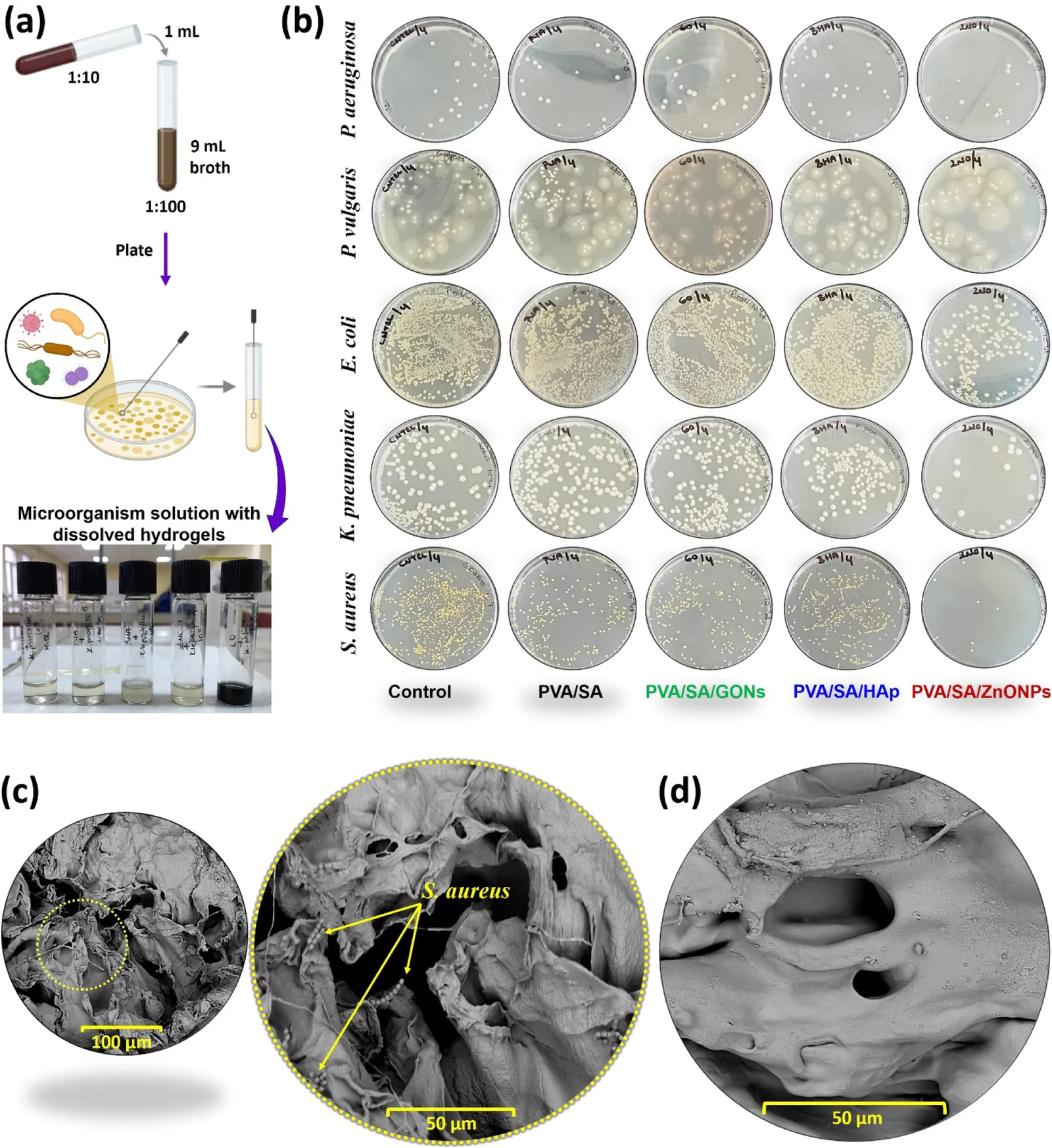
(a) Antibacterial activity evaluation test of hydrogel scaffolds, (b) Real photographs of *S. aureus*, *K. pneumoniae*, *E. coli*, *P. vulgaris* and *P. aeruginosa* grown on agar plates after different hydrogel scaffolds treatments, respectively, FE-SEM photographs of the (c) PVA/SA hydrogel scaffolds and (d) PVA/SA/ZnONPs hydrogel scaffolds, taken after 24 h at RT, show the natural proliferation of *S. aureus* microorganisms.

A fixed low nanoparticle loading of 0.1 wt % was intentionally selected as a conservative, safety-oriented formulation strategy to minimize possible concentration-dependent cytotoxic and hemolytic effects while still allowing the contribution of each nanoparticle to be compared within the same PVA/SA matrix. This is particularly important for ZnONPs, HAp, and GONs because nanoparticle biological responses are strongly dependent on dose, surface accessibility, morphology, and interactions with cells. For ZnO-based materials, Papavlassopoulos et al. reported that fibroblast viability decreased with increasing ZnO tetrapod concentration, and that direct particle–cell contact increased toxicity compared with indirect exposure through released zinc ions.^78^ Similarly, Sirelkhatim et al. reviewed that ZnO-NP bioactivity and toxicity are governed by concentration, particle size, surface area, ROS generation, Zn^2+^ release, and direct contact with biological membranes.^79^ Therefore, using 0.1 wt % ZnONPs in the present scaffold was intended to reduce excessive Zn^2+^ /ROS-mediated cellular stress while preserving a measurable antibacterial contribution. This low loading also helps explain why the antibacterial effect was moderate rather than complete, since stronger ZnO-containing wound-dressing systems have used higher ZnO contents; for example, Schadte et al. used 5 and 15 wt % tetrapodal ZnO in alginate-based wound-dressing inks and also noted that 0.5 wt % ZnO had previously produced significant antibacterial activity.^80^ Thus, the present 0.1 wt % loading represents a deliberate compromise between biological safety and antibacterial functionality, allowing nanoparticle-dependent structure–biofunction differences to be compared without overloading the hydrogel network.

### Hemolytic Activity of Hydrogel Scaffolds

Hemolytic activity is an important parameter to consider when evaluating the biocompatibility of a material, as the destruction of RBCs can cause a number of physiological problems, including anemia and inflammation. The hemolysis mechanism is shown schematically in Figure 10a, where hydrogel scaffolds interact with RBCs. The American Society for Testing and Materials (ASTM) standards state that for a material to be considered nonhemolytic, its hemolytic activity must be less than 2%. The definitions of the hemolytic index and hemolytic grade, as determined by the standards for assessing the compatibility of biomaterials with RBCs, are presented in a visual table in Figure 10b. Figure 10c illustrates the color variations among the all sample groups, the (+) control group, and the (−) control group. All four sample groups exhibited a light yellow hue, similar to the negative control group, whereas the positive control group displayed a bright red color. The hemolytic activity of the following hydrogels was evaluated, and the results are presented in Figure 10d. PVA/SA/ZnONPs has a hemolytic activity of 1.799% ± 0.01, PVA/SA has a hemolytic activity of 3.373% ± 0.04, PVA/SA/HAp has a hemolytic activity of 1.919% ± 0.04 and PVA/SA/GONs has a hemolytic activity of 6.348% ± 0.02. As per the ASTM standards, only ZnONPs and HAp reinforced hydrogel scaffolds are considered nonhemolytic as their percentage is less than 2%. PVA/SA hydrogel scaffolds are considered slightly hemolytic, while PVA/SA/GONs hydrogel scaffolds are classified as hemolytic, as their hemolytic activity ranges between 2 and 5% and exceeds 5%, respectively. The lower hemolytic activity of samples with ZnONPs and HAp may be due to the fact that these hydrogels do not have a strong ability to interact with RBCs and cause their destruction. On the other hand, GONs-containing hydrogel scaffolds may have a higher ability to interact with RBCs, which may lead to their destruction and cause hemolysis. Overall, the hemolytic activity of matrix hydrogel scaffolds and hydrogel scaffolds with ZnONPs, and HAp are acceptable and considered slightly hemolytic/nonhemolytic according to ASTM standards, while hydrogel scaffolds with GONs have high hemolytic activity and are considered hemolytic.

**Figure 10:**
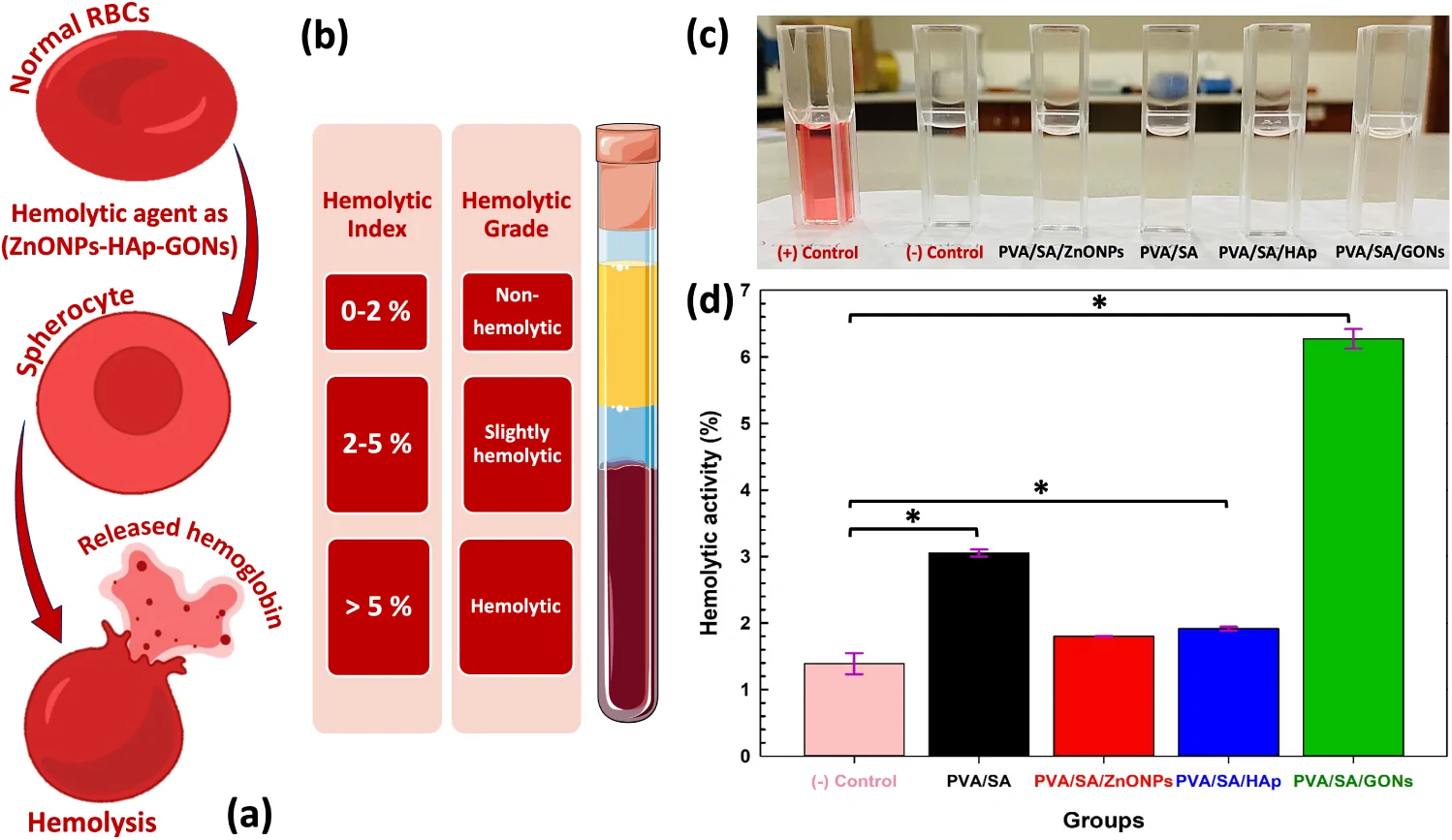
(a) Schematic representation of the hemolysis mechanism resulting from interaction with hydrogel scaffolds, (b) Hemolytic index and hemolytic grade standards data, (c) Photographs from the hemolytic activity test of all sample groups, (d) Hemolytic activity (%) of all sample groups. All data are presented as the mean (standard deviation, ±SD). (*p < 0.05; * Statistically significant differences between ZnONPs-HAp-GONs reinforced PVA/SA hydrogel scaffolds and control groups). Certain schematic elements in this figure were provided by Servier Medical Art (https://smart.servier.com), licensed under CC BY 4.0 (https://creativecommons.org/licenses/by/4.0/).

### Evaluation of Antithrombogenic Properties

The BCI is a measure of a biomaterial’s ability to prevent blood clotting. The BCI is determined by spectrophotometric measurement of the amount of free hemoglobin formed in the medium because of the contact of the relevant biomaterial with whole blood. The amount of free hemoglobin is used as a marker for clot formation. The higher the amount of free hemoglobin, the lower the clotting tendency of the biomaterial, and the higher the BCI value. The use of hydrogels in medical and biological fields is rapidly increasing due to their biocompatibility, biodegradability, and ability to mimic the extracellular matrix. In this context, the BCI of hydrogels is an important parameter to consider, as it can provide insight into the hydrogel’s ability to prevent clot formation. In the study provided, the BCI of hydrogel scaffolds at 5 min were evaluated. A detailed, enlarged visual illustration of the blood clotting process in vivo is provided in Figure 11a and the procedural steps^81,82^ and visual results of the antithrombogenic test for different hydrogel scaffold groups is depicted in Figure 11b for the comparison of hemolytic activity across the all-hydrogel scaffolds. The antithrombogenic test results in Figure 11c show that PVA/SA hydrogel scaffold has a high antithrombogenic characteristic, with BCI values of 34.22% at 5 min. This suggests that PVA/SA hydrogel scaffold has a moderate ability to prevent clot formation. In contrast, the PVA/SA/GO hydrogel scaffold exhibited the lowest BCI value (8.7% at 5 min), indicating greater clot formation on the scaffold surface under these conditions. The PVA/SA/ZnO and PVA/SA/HAp hydrogel scaffolds showed higher BCI values (54.89% and 52.74% at 5 min, respectively), indicating reduced clot formation compared with the GO-reinforced scaffold. These values suggest that the BCI% values tend to vary with the type of reinforcement agent used, indicating that the hydrogel scaffold’s ability to prevent clot formation is affected by the specific nanoparticles incorporated. Overall, higher BCI indicates lower clotting tendency under these conditions; therefore, PVA/SA/ZnO and PVA/SA/HAp showed lower clotting tendency (higher BCI), PVA/SA was intermediate, and PVA/SA/GO showed the highest clotting tendency (lowest BCI).

**Figure 11:**
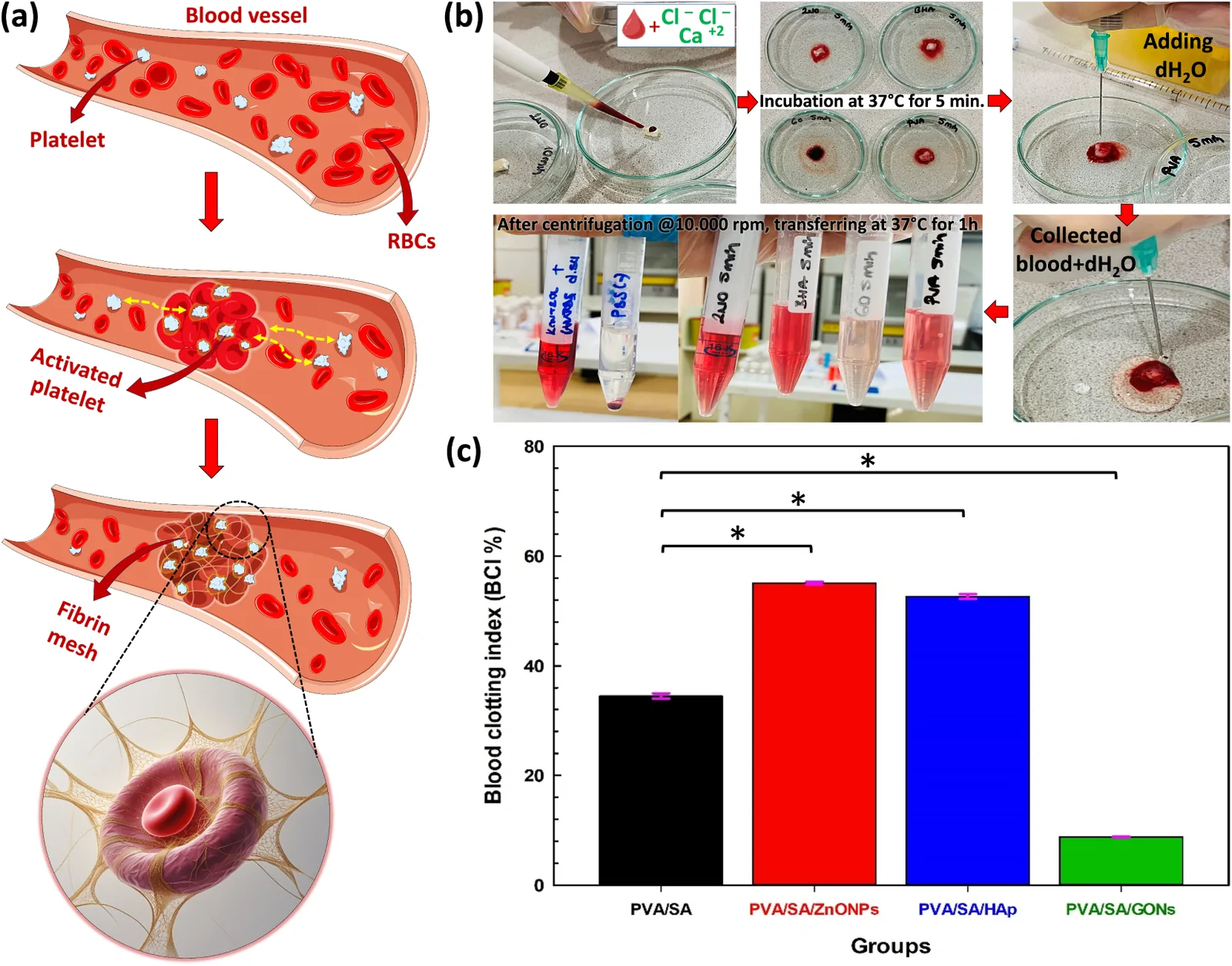
(a) The schematic representation of the blood clotting mechanism occurring as a result of biomaterial interaction. The sequence begins with platelets and RBCs in a blood vessel, followed by the activation of platelets upon contact with the biomaterial. This leads to the formation of a fibrin mesh, encapsulating the RBCs and demonstrating the clot formation process. (b) The steps and visual photographs of the blood clotting index (BCI) assay for all hydrogel scaffold groups. (c) BCI (%) results for all hydrogel scaffold groups. All data are presented as mean ± SD (*n* = 3). *p ≤ 0.05. Certain schematic elements in this figure were provided by Servier Medical Art (https://smart.servier.com), licensed under CC BY 4.0 (https://creativecommons.org/licenses/by/4.0/).

## Conclusions

This study systematically evaluated nanoparticle-reinforced PVA/SA hydrogels prepared by freeze–thaw processing to clarify filler-dependent biofunctional in tissue-contact applications. Hydrogel scaffolds were prepared using freeze–thaw processing and reinforced ZnONPs, HAp, or GONs. The scaffolds were systematically evaluated for swelling behavior, in vitro biodegradation, antibacterial performance, hemocompatibility, and cytocompatibility. Results showed that swelling ratios varied across formulations, with maximum values of 3.70 for PVA/SA, 2.46 for PVA/SA/ZnONPs, 3.39 for PVA/SA/HAp, and 3.20 for PVA/SA/GONs. Biodegradation after 28 days reached 15.9% for PVA/SA/ZnONPs, 13.1% for PVA/SA, 10.5% for PVA/SA/HAp, and 10.1% for PVA/SA/GONs. MTT assays with L929 fibroblasts confirmed high cytocompatibility, with cell viabilities of 95.3% for HAp, 91.7% for PVA/SA, 84.3% for ZnONPs, and 78.6% for GONs. Among the tested formulations, ZnONPs showed the most pronounced, although moderate, antibacterial response with a 34.6% reduction in *S. aureus* and 18.5% in *K. pneumoniae*, compared to ≤7% reduction in other groups. Hemolysis assays revealed nonhemolytic behavior for ZnONPs (1.8%) and HAp (1.9%), slightly hemolytic behavior for PVA/SA (3.4%), and hemolytic behavior for GONs (6.3%). Blood clotting index testing indicated that the GO-reinforced scaffold showed the greatest clot formation tendency (lowest BCI, 8.7%), whereas ZnO (54.9%) and HAp (52.7%) showed reduced clot formation (higher BCI %) under the tested conditions. These findings suggest that nanoparticle type significantly influences scaffold properties: ZnONPs provided moderate antibacterial protection at low concentration, HAp enhances cytocompatibility and stability, and GONs produced distinct blood-response behavior, including higher hemolysis and greater clotting tendency (lower BCI) under the tested conditions. Collectively, this work demonstrates the potential of nanoparticle-modified PVA/SA hydrogels as tunable scaffolds for specific tissue or wound healing applications.

## References

[1] Spicer C. D. (2020). Hydrogel
Scaffolds for Tissue Engineering: The Importance of Polymer Choice. Polym. Chem..

[2] Xu F., Dawson C., Lamb M., Mueller E., Stefanek E., Akbari M., Hoare T. (2022). Hydrogels for Tissue Engineering:
Addressing Key Design Needs Toward Clinical Translation. Front. Bioeng. Biotechnol..

[3] Radulescu D.-M., Neacsu I. A., Grumezescu A.-M., Andronescu E. (2022). New Insights
of Scaffolds Based on Hydrogels in Tissue Engineering. Polymers.

[4] Tang Y., Xu H., Wang X., Dong S., Guo L., Zhang S., Yang X., Liu C., Jiang X., Kan M., Wu S., Zhang J., Xu C. (2023). Advances in Preparation and Application
of Antibacterial Hydrogels. J. Nanobiotechnol..

[5] Mukasheva F., Adilova L., Dyussenbinov A., Yernaimanova B., Abilev M., Akilbekova D. (2024). Optimizing
Scaffold Pore Size for
Tissue Engineering: Insights across Various Tissue Types. Front. Bioeng. Biotechnol..

[6] Zhang Z., Feng Y., Wang L., Liu D., Qin C., Shi Y. (2022). A Review of Preparation Methods of Porous Skin Tissue
Engineering
Scaffolds. Mater. Today Commun..

[7] Cao H., Duan L., Zhang Y., Cao J., Zhang K. (2021). Current Hydrogel
Advances in Physicochemical and Biological Response-Driven Biomedical
Application Diversity. Signal Transduction Targeted
Ther..

[8] Nguyen H. M., Ngoc Le T. T., Nguyen A. T., Thien Le H. N., Pham T. T. (2023). Biomedical
Materials for Wound Dressing: Recent Advances and Applications. RSC Adv..

[9] Liu Q., Dai W., Gao Y., Li S., Zhao X., Jia H., Tan Y., Guo L., Fan Y., Zhang X. (2025). The Degradation Rate
of Collagen-Based Hydrogels Regulates Chondrogenic Differentiation
of Bone Marrow Mesenchymal Stem Cells. Collagen
Leather.

[10] Abuhamed N., Arslan S., Öksüz K.
E., Dinçer E. (2025). From Biopolymer
Matrix to Medicine: The Drug Delivery Dynamics of Amoxicillin-Loaded
PVA/SA/ZnONPs Hydrogels. Int. J. Polym. Mater.
Polym. Biomater..

[11] Chai W., Yang R., Zhou J., Wei Q. (2023). Performance
Comparison
of PVA/SA Composite Hydrogels for 3D Printing of Cartilage Scaffolds
with Different Compositions. J. Phys.: Conf.
Ser..

[12] Jiang Y., Hou Y., Fang J., Liu W., Zhao Y., Huang T., Cui J., Yang Y., Zhou Z. (2019). Preparation and Characterization
of PVA/SA/HA Composite Hydrogels for Wound Dressing. Int. J. Polym. Anal. Charact..

[13] Luo X. B., Ma M. F., Zhou X. J. (2016). Hydroxyapatite-Poly­(Vinyl
Alcohol)-Sodium
Alginate Porous Hydrogels with Poly­(Vinyl Alcohol) Surface Layer Used
for Articular Cartilage Repair. Mater. Sci.
Forum..

[14] Li S., Xiaowen Y., Yang Y., Liu L., Sun Y., Liu Y., Yin L., Chen Z. (2023). Osteogenic and Anti-Inflammatory
Effect of the Multifunctional Bionic Hydrogel Scaffold Loaded with
Aspirin and Nano-Hydroxyapatite. Front. Bioeng.
Biotechnol..

[15] Li X., Qin J., Ma J. (2015). Silk Fibroin/Poly
(Vinyl Alcohol) Blend Scaffolds for
Controlled Delivery of Curcumin. Regener. Biomater..

[16] Delgado Y.
D. L. S., Sánchez S. S., Barajas G. G. E., Quiñonez E. L., Aragón L. A. B. (2022). A Chitosan-Based
Hydrogel with PLCL, ZnO NPs, and Oligoelements:
A Promising Antibiotic Scaffold for Tissue Engineering. Rev. Mex. Ing. Bioméd..

[17] Sani E. S., Portillo-Lara R., Spencer A., Yu W., Geilich B. M., Noshadi I., Webster T. J., Annabi N. (2018). Engineering Adhesive
and Antimicrobial Hyaluronic Acid/Elastin-like Polypeptide Hybrid
Hydrogels for Tissue Engineering Applications. ACS Biomater. Sci. Eng..

[18] Senapati S., Mahanta A. K., Kumar S., Maiti P. (2018). Controlled Drug Delivery
Vehicles for Cancer Treatment and Their Performance. Signal Transduction Targeted Ther..

[19] Öksüz K.
E. (2024). Bioactive
Coatings on Biopolymer Materials: Evaluation of Mechanical, Physical,
Thermal, and in Vitro Properties. J. Aust. Ceram.
Soc..

[20] You T., You Q., Feng X., Li H., Yi B., Xu H. (2024). A Novel Approach
to Wound Healing: Green Synthetic Nano-Zinc Oxide Embedded with Sodium
Alginate and Polyvinyl Alcohol Hydrogels for Dressings. Int. J. Pharm..

[21] Öksüz K. E., Kilinç S., Özer A. (2019). Effect of Calcination on Microstructure
Development and Properties of Hydroxyapatite Powders Extracted from
Human and Bovine Bones. Trans. Indian Ceram.
Soc..

[22] Özer A., Öksüz K.
E. (2019). The Effect of Yttrium
Oxide in Hydroxyapatite/Aluminum
Oxide Hybrid Biocomposite Materials: Phase, Mechanical and Morphological
Evaluation. Materialwiss. Werkstofftech..

[23] Gunduz O., Gode C., Ahmad Z., Gökçe H., Yetmez M., Kalkandelen C., Sahin Y. M., Oktar F. N. (2014). Preparation
and Evaluation of Cerium Oxide-Bovine Hydroxyapatite Composites for
Biomedical Engineering Applications. J. Mech.
Behav. Biomed. Mater..

[24] Chu S.-Y., Yan T.-M., Chen S.-L. (2000). Characteristics
of Sol-Gel Synthesis
of ZnO-Based Powders. J. Mater. Sci. Lett..

[25] Azam A., Ahmed F., Arshi N., Chaman M., Naqvi A. H. (2010). Formation
and Characterization of ZnO Nanopowder Synthesized by Sol–Gel
Method. J. Alloys Compd..

[26] Lin Y.-C., Cao Y., Jang J.-H., Shu C.-M., Webb C., Pan W.-P. (2014). The Synthesis
and Characterization of Graphene Oxides Based on a Modified Approach. J. Therm. Anal. Calorim..

[27] Öksüz K.
E., Özkaya N. K., İnan Z. D., Özer A. (2021). Novel Natural
Spider Silk Embedded Electrospun Nanofiber Mats for Wound Healing. Mater. Today Commun..

[28] Cottam E., Hukins D. W. L., Lee K., Hewitt C., Jenkins M. J. (2009). Effect
of Sterilisation by Gamma Irradiation on the Ability of Polycaprolactone
(PCL) to Act as a Scaffold Material. Med. Eng.
Phys..

[29] Demirkiran B. B., Inan Z. D. S., HamutoĞlu R., Öksüz K. E., Hasbek Z., Altuntaş E. E. (2025). Boron-Doped
Nano Hydroxyapatite Grafts
for Bone Regeneration in Rat Mandibular Defects. Biol. Trace Elem. Res..

[30] Rebia R. A., Rozet S., Tamada Y., Tanaka T. (2018). Biodegradable PHBH/PVA
Blend Nanofibers: Fabrication, Characterization, in Vitro Degradation,
and in Vitro Biocompatibility. Polym. Degrad.
Stab..

[31] Zhao H., Li Y. (2020). A Novel PH/Temperature-Responsive Hydrogel Based on Tremella Polysaccharide
and Poly­(N-Isopropylacrylamide). Colloids Surf.,
A.

[32] Öksüz K.
E., Kurt B., İnan Z. D. N., Hepokur C. (2023). Novel Bioactive Glass/Graphene
Oxide-Coated Surgical Sutures for Soft Tissue Regeneration. ACS Omega.

[33] Patil P. P., Meshram J. V., Bohara R. A., Nanaware S. G., Pawar S. H. (2018). ZnO Nanoparticle-Embedded
Silk Fibroin–Polyvinyl Alcohol Composite Film: A Potential
Dressing Material for Infected Wounds. New J.
Chem..

[34] Bohorquez-Moreno C. D., Öksüz K. E., Dinçer E. (2023). Porous Polymer
Scaffolds Derived from Bioresources for Biomedical Applications. Cellul. Chem. Technol..

[35] Ateş A., Aydemir B., Öksüz K.
E. (2024). Investigation of Physicochemical
and Biological Properties of Boron-Doped Biochar. Biomass Convers. Biorefin..

[36] Gao L., Chen J., Feng W., Song Q., Huo J., Yu L., Liu N., Wang T., Li P., Huang W. (2020). A Multifunctional
Shape-Adaptive and Biodegradable Hydrogel with Hemorrhage Control
and Broad-Spectrum Antimicrobial Activity for Wound Healing. Biomater. Sci..

[37] Mohanta Y. K., Biswas K., Rauta P. R., Mishra A. K., De D., Hashem A., Al-Arjani A.-B. F., Alqarawi A. A., Abd-Allah E. F., Mahanta S., Mohanta T. K. (2021). Development
of Graphene Oxide Nanosheets
as Potential Biomaterials in Cancer Therapeutics: An In-Vitro Study
Against Breast Cancer Cell Line. J. Inorg. Organomet.
Polym. Mater..

[38] Wang J., Wang Y., Zhang Y., Uliana A., Zhu J., Liu J., Van Der Bruggen B. (2016). Zeolitic Imidazolate Framework/Graphene
Oxide Hybrid Nanosheets Functionalized Thin Film Nanocomposite Membrane
for Enhanced Antimicrobial Performance. ACS
Appl. Mater. Interfaces.

[39] Arthanari S., Mani G., Jang J. H., Choi J. O., Cho Y. H., Lee J. H., Cha S. E., Oh H. S., Kwon D. H., Jang H. T. (2016). Preparation and
Characterization of Gatifloxacin-Loaded
Alginate/Poly (Vinyl Alcohol) Electrospun Nanofibers. Artif. Cells, Nanomed., Biotechnol..

[40] Raza M. A., Islam A., Sabir A., Gull N., Ali I., Mehmood R., Bae J., Hassan G., Khan M. U. (2019). PVA/TEOS
Crosslinked Membranes Incorporating Zinc Oxide Nanoparticles and Sodium
Alginate to Improve Reverse Osmosis Performance for Desalination. J. Appl. Polym. Sci..

[41] Sarkar R., Ghosh A., Barui A., Datta P. (2018). Repositing Honey Incorporated
Electrospun Nanofiber Membranes to Provide Anti-Oxidant, Anti-Bacterial
and Anti-Inflammatory Microenvironment for Wound Regeneration. J. Mater. Sci.: Mater. Med..

[42] Prakash J., Kumar T. S., Venkataprasanna K. S., Niranjan R., Kaushik M., Samal D. B., Venkatasubbu G. D. (2019). PVA/Alginate/Hydroxyapatite
Films
for Controlled Release of Amoxicillin for the Treatment of Periodontal
Defects. Appl. Surf. Sci..

[43] Mahmood A., Bano S., Kim S.-G., Lee K.-H. (2012). Water–Methanol
Separation Characteristics of Annealed SA/PVA Complex Membranes. J. Membr. Sci..

[44] Kumaraswamy S., Babaladimath G., Badalamoole V., Mallaiah S. H. (2017). Gamma Irradiation
Synthesis and in Vitro Drug Release Studies of ZnO/PVA Hydrogel Nanocomposites. Adv. Mater. Lett..

[45] Amiri S., Asghari A., Vatanpour V., Rajabi M. (2020). Fabrication and Characterization
of a Novel Polyvinyl Alcohol-Graphene Oxide-Sodium Alginate Nanocomposite
Hydrogel Blended PES Nanofiltration Membrane for Improved Water Purification. Sep. Purif. Technol..

[46] Liu C., Liu H., Xiong T., Xu A., Pan B., Tang K. (2018). Graphene Oxide
Reinforced Alginate/PVA Double Network Hydrogels for Efficient Dye
Removal. Polymers.

[47] Lin X., Zhang J., Feng H., Xue C. (2023). The Influence of Graphene
Oxide Content on Adsorption of PVA/SA Composite Gel Spheres. Colloid Polym. Sci..

[48] Thayumanavan N., Tambe P., Joshi G., Shukla M. (2014). Effect of Sodium Alginate
Modification of Graphene (by ‘Anion- π’ Type of
Interaction) on the Mechanical and Thermal Properties of Polyvinyl
Alcohol (PVA) Nanocomposites. Compos. Interfaces.

[49] Kumar K. C. V., Subha T. J., Ahila K. G., Ravindran B., Chang S. W., Mahmoud A. H., Mohammed O. B., Rathi M. A. (2021). Spectral
Characterization of Hydroxyapatite Extracted from Black Sumatra and
Fighting Cock Bone Samples: A Comparative Analysis. Saudi J. Biol. Sci..

[50] Prosanov I. Y., Matvienko A. A. (2010). Study of
PVA Thermal Destruction by Means of IR and
Raman Spectroscopy. Phys. Solid State.

[51] Shi Y., Xiong D., Li J., Wang K., Wang N. (2017). In Situ Repair
of Graphene Defects and Enhancement of Its Reinforcement Effect in
Polyvinyl Alcohol Hydrogels. RSC Adv..

[52] Zubair N.
A., Rahman N. A., Lim H. N., Zawawi R. M., Sulaiman Y. (2016). Electrochemical
Properties of PVA–GO/PEDOT Nanofibers Prepared Using Electrospinning
and Electropolymerization Techniques. RSC Adv..

[53] Feraru A., Tóth Z.-R., Mureşan-Pop M., Baia M., Gyulavári T., Páll E., Turcu R. V. F., Magyari K., Baia L. (2023). Anionic Polysaccharide
Cryogels: Interaction and In Vitro Behavior of Alginate–Gum
Arabic Composites. Polymers.

[54] Xu X., Lv C., You X., Wang B., Ji F., Hu B. (2017). Nitrogen Removal
and Microbial Diversity of Activated Sludge Entrapped in Modified
Poly­(Vinyl Alcohol)–Sodium Alginate Gel. Int. Biodeterior. Biodegrad..

[55] Pandey P., Parra M. R., Haque F. Z., Kurchania R. (2017). Effects of
Annealing Temperature Optimization on the Efficiency of ZnO Nanoparticles
Photoanode Based Dye Sensitized Solar Cells. J. Mater. Sci.: Mater. Electron..

[56] Sharma A., Singh B. P., Dhar S., Gondorf A., Spasova M. (2012). Effect of
Surface Groups on the Luminescence Property of ZnO Nanoparticles Synthesized
by Sol–Gel Route. Surf. Sci..

[57] Sekar V., Narenkumar J., Rajasekar A., Santhanam A., Velmurugan P., Khan M. M., Basavegowda N. (2024). HAP-Based
Nanocomposite and Vitamin C for Sustained Drug Delivery of Amoxicillin. BioNanoScience.

[58] Chopra H., Kumar S., Singh I. (2022). Strategies
and Therapies for Wound
Healing: A Review. Curr. Drug Targets.

[59] Xie L., Jiang M., Dong X., Bai X., Tong J., Zhou J. (2012). Controlled Mechanical and Swelling Properties of Poly­(Vinyl Alcohol)/Sodium
Alginate Blend Hydrogels Prepared by Freeze–Thaw Followed by
Ca2+ Crosslinking. J. Appl. Polym. Sci..

[60] Tantiwatcharothai S., Prachayawarakorn J. (2019). Characterization of an Antibacterial Wound Dressing
from Basil Seed (Ocimum Basilicum L.) Mucilage-ZnO Nanocomposite. Int. J. Biol. Macromol..

[61] Marvizadeh M. M., Oladzadabbasabadi N., Nafchi A. M., Jokar M. (2017). Preparation and Characterization
of Bionanocomposite Film Based on Tapioca Starch/Bovine Gelatin/Nanorod
Zinc Oxide. Int. J. Biol. Macromol..

[62] Chocholata P., Kulda V., Dvorakova J., Supova M., Zaloudkova M., Babuska V. (2021). In Situ Hydroxyapatite
Synthesis Enhances Biocompatibility
of PVA/HA Hydrogels. Int. J. Mol. Sci..

[63] Ma P., Wu W., Wei Y., Ren L., Lin S., Wu J. (2021). Biomimetic
Gelatin/Chitosan/Polyvinyl Alcohol/Nano-Hydroxyapatite Scaffolds for
Bone Tissue Engineering. Mater. Des..

[64] Al-Mofty S. E.-D., Karaly A. H., Sarhan W. A., Azzazy H. M. E. (2022). Multifunctional
Hemostatic PVA/Chitosan Sponges Loaded with Hydroxyapatite and Ciprofloxacin. ACS Omega.

[65] Xue K., Teng S.-H., Niu N., Wang P. (2018). Biomimetic Synthesis
of Novel Polyvinyl Alcohol/Hydroxyapatite Composite Microspheres for
Biomedical Applications. Mater. Res. Express.

[66] Kaur T., Thirugnanam A., Pramanik K. (2016). Tailoring the in Vitro Characteristics
of Poly­(Vinyl Alcohol)-Nanohydroxyapatite Composite Scaffolds for
Bone Tissue Engineering. J. Polym. Eng..

[67] Wang C., Hao H., Wang J., Xue Y., Huang J., Ren K., Ji J. (2021). High-Throughput Hyaluronic Acid Hydrogel Arrays for Cell Selective
Adhesion Screening. J. Mater. Chem. B.

[68] Kamoun E.
A., Kenawy E.-R. S., Tamer T. M., El-Meligy M. A., Eldin M. S. M. (2015). Poly (Vinyl Alcohol)-Alginate
Physically Crosslinked
Hydrogel Membranes for Wound Dressing Applications: Characterization
and Bio-Evaluation. Arabian J. Chem..

[69] Bahadoran M., Shamloo A., Nokoorani Y. D. (2020). Development of a Polyvinyl Alcohol/Sodium
Alginate Hydrogel-Based Scaffold Incorporating BFGF-Encapsulated Microspheres
for Accelerated Wound Healing. Sci. Rep..

[70] Dogan H. Y., Altin Y., BedeloĞlu A. Ç. (2022). Fabrication and Properties of Graphene
Oxide and Reduced Graphene Oxide Reinforced Poly­(Vinyl Alcohol) Nanocomposite
Films for Packaging Applications. Polym. Polym.
Compos..

[71] Xu M., Qin M., Zhang X., Zhang X., Li J., Hu Y., Chen W., Huang D. (2020). Porous PVA/SA/HA Hydrogels Fabricated
by Dual-Crosslinking Method for Bone Tissue Engineering. J. Biomater. Sci., Polym. Ed..

[72] Munir M. U., Mikucioniene D., Khanzada H., Khan M. Q. (2022). Development of Eco-Friendly
Nanomembranes of Aloe Vera/PVA/ZnO for Potential Applications in Medical
Devices. Polymers.

[73] Jalageri M. B., Kumar G. C. M. (2024). Graphene Oxide
Reinforced Polyvinyl Alcohol/Chitosan
Composite Hydrogel for Cartilage Regeneration. Polym. Bull..

[74] Kenawy E.-R., Kamoun E. A., Shehata A., El-Moslamy S. H., Abdel-Rahman A. A.-H. (2025). Biosynthesized ZnO NPs Loaded-Electrospun PVA/Sodium
Alginate/Glycine Nanofibers: Synthesis, Spinning Optimization and
Antimicrobial Activity Evaluation. Sci. Rep..

[75] Zhu J., Cheng H., Zhang Z., Chen K., Zhang Q., Zhang C., Gao W., Zheng Y. (2024). Antibacterial Hydrogels
for Wound Dressing Applications: Current Status, Progress, Challenges,
and Trends. Gels.

[76] Wu S., Liu Y., Zhang H., Lei L. (2019). Nano-Graphene Oxide Improved the
Antibacterial Property of Antisense YycG RNA on *Staphylococcus
Aureus*. J. Orthop. Surg..

[77] Dorozhkin S. V. (2021). Synthetic
Amorphous Calcium Phosphates (ACPs): Preparation, Structure, Properties,
and Biomedical Applications. Biomater. Sci..

[78] Papavlassopoulos H., Mishra Y. K., Kaps S., Paulowicz I., Abdelaziz R., Elbahri M., Maser E., Adelung R., Röhl C. (2014). Toxicity of Functional Nano-Micro Zinc Oxide Tetrapods:
Impact of Cell Culture Conditions, Cellular Age and Material Properties. PLoS One.

[79] Sirelkhatim A., Mahmud S., Seeni A., Kaus N. H. M., Ann L. C., Bakhori S. K. M., Hasan H., Mohamad D. (2015). Review on
Zinc Oxide
Nanoparticles: Antibacterial Activity and Toxicity Mechanism. Nanomicro Lett..

[80] Schadte P., Rademacher F., Andresen G., Hellfritzsch M., Qiu H., Maschkowitz G., Gläser R., Heinemann N., Drücke D., Fickenscher H., Scherließ R., Harder J., Adelung R., Siebert L. (2023). 3D-Printed Wound Dressing
Platform for Protein Administration Based on Alginate and Zinc Oxide
Tetrapods. Nano Convergence.

[81] Sabino R. M., Popat K. C. (2020). Evaluating Whole
Blood Clotting in Vitro on Biomaterial
Surfaces. Bio-Protoc..

[82] Pourshahrestani S., Zeimaran E., Kadri N. A., Mutlu N., Boccaccini A. R. (2020). Polymeric
Hydrogel Systems as Emerging Biomaterial Platforms to Enable Hemostasis
and Wound Healing. Adv. Healthcare Mater..

